# Supplementary material for: S100A4+ macrophages facilitate zika virus invasion and persistence in the seminiferous tubules via interferon-gamma mediation
Source: PLoS Pathog. 2020 Dec 14;16(12):e1009019. doi: 10.1371/journal.ppat.1009019 (PMC7769614; doi:10.1371/journal.ppat.1009019)
Supplement: S1 Table — Transcriptome of ZIKV-infected AG6 testes was analyzed by RNA-sequencing. All up-regulated genes were showed in this table. (PDF) [file ppat.1009019.s001.pdf]

S1 Table. Up-regulated genes in testicles of ZIKV-infected AG6 mice at 5 dpi

Gene Name	log2 (Fold Change)	Fold Change	p value[a]	q value[b]	Control Testis FPKM	Test Testis FPKM	ENSEMBL_GeneID
Saa3	8.09	273.26	0.00	0.00	1.14	311.18	ENSMUSG00000040026.7
Cacul1, Gm25238	5.47	44.23	0.00	0.02	1.40	61.76	ENSMUSG00000092655.1
S100a8	5.43	12.42	0.01	0.14	0.37	16.02	ENSMUSG00000056054.9
Spp1	3.48	11.17	0.00	0.00	2.48	27.70	ENSMUSG00000029304.14
Ccl2	4.99	8.98	0.00	0.01	0.35	11.12	ENSMUSG00000035385.5
Ccl5	3.05	8.27	0.00	0.08	1.55	12.86	ENSMUSG00000035042.2
S100a4	2.51	5.70	0.00	0.02	5.83	33.19	ENSMUSG00000105518.1
Timp1	2.28	4.87	0.04	0.41	4.80	23.38	ENSMUSG00000001131.11
Lilr4b, Lilrb4a	2.24	4.72	0.00	0.06	1.23	5.79	ENSMUSG00000089672.4
Coro1a	2.17	4.51	0.02	0.33	1.91	8.63	ENSMUSG00000030707.15
Oasl2	2.14	4.42	0.00	0.02	1.27	5.61	ENSMUSG00000029561.17
Serpina3n	2.09	4.25	0.00	0.01	5.62	23.87	ENSMUSG00000021091.8
C3	2.08	4.24	0.00	0.00	17.36	73.66	ENSMUSG00000024164.15
Arg1	5.11	4.04	0.00	0.01	0.10	3.44	ENSMUSG00000019987.8
Serpina3g	1.91	3.75	0.01	0.18	3.12	11.70	ENSMUSG00000041481.16
Ifit3	3.60	3.74	0.00	0.07	0.33	3.97	ENSMUSG00000074896.3
Ccl7	3.28	3.62	0.01	0.17	0.43	4.19	ENSMUSG00000035373.2
Wisp2	2.60	3.31	0.00	0.03	0.83	5.06	ENSMUSG00000027656.7
Traf1	1.71	3.28	0.02	0.27	1.08	3.54	ENSMUSG00000026875.14
Cd96	1.71	3.27	0.05	0.44	11.23	36.78	ENSMUSG00000022657.9
Gm4758	1.69	3.22	0.03	0.33	3.09	9.96	ENSMUSG00000079595.1
4931408C20Rik	1.62	3.08	0.01	0.14	3.42	10.53	ENSMUSG00000073722.3
Msr1	1.60	3.03	0.02	0.32	3.41	10.33	ENSMUSG00000025044.15
Hemgn	1.58	2.99	0.00	0.07	19.24	57.43	ENSMUSG00000028332.13
Spata31d1c	1.57	2.98	0.00	0.12	7.71	22.98	ENSMUSG00000074849.2
Fam71b	1.56	2.94	0.01	0.16	40.26	118.49	ENSMUSG00000020401.6
Olfr128	1.56	2.94	0.04	0.43	10.11	29.72	ENSMUSG00000059030.4
Asap3	1.55	2.92	0.01	0.18	1.71	4.99	ENSMUSG00000036995.7
Fam71a	1.54	2.91	0.00	0.06	23.46	68.22	ENSMUSG00000091017.1
Trim42	1.54	2.91	0.01	0.14	7.67	22.29	ENSMUSG00000032451.6
Chtf18	1.53	2.89	0.01	0.14	3.39	9.81	ENSMUSG00000019214.13
Rac2	1.52	2.88	0.03	0.34	1.01	2.91	ENSMUSG00000033220.6
Bcl11a	1.52	2.87	0.03	0.37	1.93	5.56	ENSMUSG00000000861.15
4921508M14Rik, Hdac9	1.52	2.87	0.04	0.41	7.43	21.32	ENSMUSG00000052376.2
Hhipl2	1.51	2.86	0.01	0.18	1.70	4.87	ENSMUSG00000053461.9
Actrt3	1.51	2.85	0.02	0.28	12.97	36.97	ENSMUSG00000037737.6
Iqcf6	1.51	2.84	0.01	0.20	28.07	79.82	ENSMUSG00000091129.2
Gm8251, Mettl21c	1.51	2.84	0.01	0.23	5.56	15.79	ENSMUSG00000091844.1
Tmem151a	1.51	2.84	0.04	0.43	7.32	20.79	ENSMUSG00000061451.13
Myo15	1.50	2.83	0.01	0.25	1.32	3.73	ENSMUSG00000042678.16
Prss52	1.49	2.81	0.01	0.25	26.47	74.47	ENSMUSG00000021966.7

Ccdc54	1.49	2.80	0.00	0.09	95.47	267.67	ENSMUSG00000050685.4
Baz1a,Gm20403	1.48	2.79	0.00	0.08	21.88	61.11	ENSMUSG00000035021.13
Spata31d1b	1.48	2.79	0.00	0.05	22.17	61.84	ENSMUSG00000091311.2
Fbxo39	1.48	2.78	0.01	0.18	18.27	50.85	ENSMUSG00000070388.3
Kif5c	1.47	2.78	0.02	0.31	5.14	14.29	ENSMUSG00000026764.15
Ccdc185	1.47	2.77	0.01	0.15	47.43	131.56	ENSMUSG00000043429.7
Gm28269	1.47	2.76	0.01	0.14	23.51	64.99	ENSMUSG00000101818.1
Vmn2r111	1.46	2.76	0.01	0.15	5.87	16.20	ENSMUSG00000095093.1
Ccin	1.46	2.75	0.01	0.19	59.43	163.14	ENSMUSG00000070999.2
D930020B18Rik	2.46	2.74	0.03	0.39	0.63	3.48	ENSMUSG00000047642.14
Tuba8	1.45	2.74	0.01	0.16	49.56	135.82	ENSMUSG00000030137.8
Saxo1	1.45	2.74	0.01	0.16	25.53	69.95	ENSMUSG00000028492.13
Ms4a6d	2.98	2.73	0.00	0.07	0.34	2.65	ENSMUSG00000024679.10
Aknad1	1.45	2.73	0.01	0.19	6.40	17.49	ENSMUSG00000049565.16
BC049730	1.44	2.72	0.00	0.13	17.80	48.35	ENSMUSG00000045587.9
Gm4922	1.44	2.71	0.03	0.35	3.25	8.81	ENSMUSG00000044624.6
Gm136	1.43	2.70	0.00	0.11	26.29	70.89	ENSMUSG00000071015.2
Srgap1	1.43	2.69	0.05	0.47	6.38	17.18	ENSMUSG00000020121.15
Nr5a1	1.43	2.69	0.01	0.17	4.48	12.07	ENSMUSG00000026751.14
Kcnip2	1.43	2.69	0.01	0.23	3.58	9.62	ENSMUSG00000025221.15
Adora3, Tmigd3	1.43	2.69	0.01	0.16	49.44	132.86	ENSMUSG00000000562.5
Scp2d1	1.42	2.68	0.00	0.06	125.00	335.47	ENSMUSG00000027431.5
Gm5449	1.42	2.68	0.03	0.35	2.63	7.06	ENSMUSG00000063166.4
Ces1h	1.42	2.68	0.01	0.15	3.41	9.13	ENSMUSG00000074156.4
1700016C15Rik	1.41	2.66	0.02	0.32	43.14	114.96	ENSMUSG00000015962.5
Zfp536	1.41	2.66	0.01	0.25	1.00	2.67	ENSMUSG00000043456.16
Vcam1	1.41	2.66	0.00	0.08	9.97	26.54	ENSMUSG00000027962.14
Exo1	1.40	2.65	0.02	0.29	1.96	5.17	ENSMUSG00000039748.11
Pappa	1.40	2.64	0.01	0.20	1.07	2.82	ENSMUSG00000028370.7
Myt1l	1.40	2.64	0.04	0.41	1.47	3.88	ENSMUSG00000061911.14
Wbp2nl	1.40	2.63	0.00	0.11	23.27	61.32	ENSMUSG00000022455.3
Noxred1	1.40	2.63	0.01	0.14	20.26	53.30	ENSMUSG00000072919.3
Clec4g	1.39	2.63	0.02	0.28	3.31	8.70	ENSMUSG00000074491.9
4933427I04Rik	1.39	2.63	0.02	0.33	14.92	39.18	ENSMUSG00000073761.2
Klf17	1.39	2.62	0.01	0.22	5.90	15.49	ENSMUSG00000048626.5
Mvk	inf	2.62	0.04	0.43	0.00	1.62	ENSMUSG00000041939.14
Myo18b	1.39	2.62	0.02	0.32	12.13	31.77	ENSMUSG00000072720.9
Nkg7	2.84	2.60	0.01	0.15	0.35	2.53	ENSMUSG00000004612.9
Spata31d1a	1.38	2.60	0.01	0.17	13.11	34.13	ENSMUSG00000050876.8
Zfp37	1.37	2.59	0.02	0.31	29.04	75.24	ENSMUSG00000028389.10
Klrb1	1.37	2.59	0.02	0.28	9.67	25.01	ENSMUSG00000079299.3
Cstl1	1.37	2.58	0.03	0.33	58.94	152.19	ENSMUSG00000055177.15
4921528I07Rik	1.37	2.58	0.01	0.23	12.48	32.22	ENSMUSG00000064299.5
Gm5878	1.37	2.58	0.01	0.21	5.27	13.60	ENSMUSG00000072952.6
Tmco5b	1.37	2.58	0.01	0.20	9.43	24.30	ENSMUSG00000041255.2
4930435E12Rik	1.36	2.57	0.02	0.27	16.73	43.05	ENSMUSG00000022798.7
Cntn1	1.36	2.57	0.02	0.33	2.12	5.45	ENSMUSG00000055022.14

Prss58	1.36	2.57	0.01	0.20	24.86	63.82	ENSMUSG00000051936.4
Mex3b	1.36	2.57	0.01	0.24	8.27	21.23	ENSMUSG00000030636.15
Smim23	1.36	2.56	0.01	0.25	77.42	198.50	ENSMUSG00000020270.9
Gata1	1.36	2.56	0.02	0.30	2.10	5.39	ENSMUSG00000031162.14
Cypt2	1.36	2.56	0.01	0.15	34.95	89.46	ENSMUSG00000090132.2
4930516K23Rik	1.36	2.56	0.00	0.12	19.08	48.82	ENSMUSG00000066263.8
4930407I10Rik	1.35	2.56	0.01	0.19	37.88	96.88	ENSMUSG00000075524.2
Prss55	1.35	2.56	0.03	0.35	56.31	144.00	ENSMUSG00000034623.14
Prox1	1.35	2.56	0.04	0.40	1.81	4.62	ENSMUSG00000010175.13
4933412E24Rik	1.35	2.55	0.02	0.33	4.24	10.82	ENSMUSG00000071749.2
Cntnap5b	1.35	2.55	0.01	0.20	17.79	45.33	ENSMUSG00000067028.11
Cypt4	1.35	2.55	0.03	0.36	218.98	557.78	ENSMUSG00000047995.6
Tex29	1.35	2.55	0.01	0.20	78.59	200.03	ENSMUSG00000031512.14
Ttll2	1.35	2.54	0.02	0.28	5.78	14.71	ENSMUSG00000079722.1
Cypt1	1.35	2.54	0.01	0.15	83.01	211.14	ENSMUSG00000023257.3
Pcsk1n	1.35	2.54	0.04	0.40	1.64	4.18	ENSMUSG00000039278.10
F5	1.34	2.54	0.02	0.29	1.56	3.97	ENSMUSG00000026579.8
1700008P02Rik	1.34	2.53	0.01	0.20	17.63	44.63	ENSMUSG00000069118.3
Gjd3	1.34	2.53	0.04	0.43	1.73	4.36	ENSMUSG00000047197.1
Gm5174	1.33	2.52	0.01	0.25	3.67	9.27	ENSMUSG00000090308.1
Susd5	1.33	2.52	0.02	0.32	1.42	3.58	ENSMUSG00000086596.1
Fam107b	1.33	2.51	0.02	0.30	41.53	104.30	ENSMUSG00000026655.14
Ccl9	2.01	2.51	0.01	0.24	0.99	3.98	ENSMUSG00000019122.8
Clec12b	1.33	2.51	0.05	0.44	2.50	6.27	ENSMUSG00000030158.12
1700129C05Rik	1.32	2.50	0.01	0.24	127.61	319.34	ENSMUSG00000021977.11
Spanxn4	1.32	2.50	0.01	0.15	80.60	201.49	ENSMUSG00000091396.1
Adam6a, Adam6b	1.32	2.50	0.00	0.10	21.81	54.52	ENSMUSG00000043945.4
Mroh3	1.32	2.50	0.04	0.40	2.70	6.75	ENSMUSG00000087230.8
Wdr64	1.32	2.50	0.01	0.21	32.76	81.81	ENSMUSG00000026523.14
Aqp7	1.32	2.50	0.01	0.18	27.67	69.09	ENSMUSG00000028427.13
Smco2	1.32	2.49	0.00	0.13	44.62	111.23	ENSMUSG00000030292.10
Cct8l1	1.32	2.49	0.01	0.13	20.71	51.61	ENSMUSG00000038044.8
Diaph3	1.32	2.49	0.02	0.29	8.62	21.46	ENSMUSG00000022021.13
Sppl2c	1.31	2.49	0.01	0.16	69.25	172.17	ENSMUSG00000049506.7
Srcin1	1.31	2.48	0.04	0.42	15.41	38.28	ENSMUSG00000038453.16
Trim80	1.31	2.48	0.01	0.20	50.72	125.99	ENSMUSG00000070332.4
4921530L21Rik	1.31	2.48	0.01	0.17	29.26	72.68	ENSMUSG00000034689.1
Gm382	1.31	2.48	0.01	0.23	1.82	4.51	ENSMUSG00000062791.4
Asb9	1.31	2.48	0.01	0.19	21.66	53.71	ENSMUSG00000031384.2
Unc13d	1.31	2.48	0.05	0.47	1.06	2.63	ENSMUSG00000057948.12
Cypt12	1.31	2.47	0.02	0.26	379.11	937.59	ENSMUSG00000027564.4
Tekt5	1.31	2.47	0.01	0.21	42.12	104.08	ENSMUSG00000039179.13
Fam181a	1.30	2.47	0.04	0.44	16.35	40.37	ENSMUSG00000096753.7
Gm14474	1.30	2.47	0.03	0.36	38.49	94.93	ENSMUSG00000096097.1
Fam71f1	1.30	2.47	0.01	0.23	80.51	198.49	ENSMUSG00000039742.15
Gm5382	1.30	2.46	0.01	0.20	27.69	68.20	ENSMUSG00000078346.3
Prss35	1.30	2.46	0.03	0.36	1.71	4.21	ENSMUSG00000033491.13

Esco2	1.30	2.46	0.01	0.18	1.78	4.37	ENSMUSG00000022034.9
Gm527	1.30	2.46	0.01	0.17	11.06	27.18	ENSMUSG00000047227.4
Sox30	1.29	2.45	0.04	0.42	34.17	83.85	ENSMUSG00000040489.5
Micalcl	1.29	2.45	0.01	0.21	43.25	106.03	ENSMUSG00000030771.15
Ksr1	1.29	2.45	0.04	0.39	1.25	3.07	ENSMUSG00000018334.17
Tmprss12	1.29	2.45	0.03	0.36	5.75	14.09	ENSMUSG00000045631.9
Ctnna3	1.29	2.45	0.02	0.31	13.24	32.42	ENSMUSG00000060843.11
Chga	1.29	2.45	0.04	0.39	2.28	5.58	ENSMUSG00000021194.5
Rnf138rt1	1.29	2.45	0.01	0.22	7.34	17.97	ENSMUSG00000083695.6
4921507P07Rik	1.29	2.45	0.02	0.32	63.69	155.84	ENSMUSG00000029828.4
Banf2	1.29	2.44	0.01	0.25	61.74	150.89	ENSMUSG00000037307.11
Fhdc1	1.29	2.44	0.01	0.18	9.60	23.43	ENSMUSG00000041842.15
Cdyl	1.29	2.44	0.00	0.12	40.09	97.82	ENSMUSG00000059288.13
Cylc1	1.29	2.44	0.01	0.18	15.40	37.53	ENSMUSG00000073001.2
1700019O17Rik	1.29	2.44	0.02	0.27	27.98	68.20	ENSMUSG00000036574.5
Hecw1	1.28	2.44	0.04	0.43	3.61	8.79	ENSMUSG00000021301.8
Cap2	1.28	2.43	0.01	0.19	12.85	31.25	ENSMUSG00000021373.15
Tex33	1.28	2.43	0.04	0.44	227.51	553.34	ENSMUSG00000062154.13
Hils1	1.28	2.42	0.02	0.27	301.99	731.00	ENSMUSG00000038994.6
Actrt2	1.27	2.42	0.01	0.25	139.79	338.28	ENSMUSG00000051276.4
Efcab3, Gm11639	1.27	2.42	0.02	0.32	55.97	135.40	ENSMUSG00000040838.9
Asb17	1.27	2.42	0.04	0.40	56.30	136.17	ENSMUSG00000038997.4
Plbd1	1.27	2.42	0.01	0.14	10.67	25.80	ENSMUSG00000030214.6
Lexm	1.27	2.42	0.04	0.42	18.71	45.23	ENSMUSG00000054362.9
Dpysl5, Mapre3	1.27	2.41	0.02	0.31	66.93	161.55	ENSMUSG00000029166.14
Dmc1	1.27	2.41	0.03	0.37	2.39	5.75	ENSMUSG00000022429.10
Gtsf1l	1.27	2.41	0.01	0.22	189.74	456.49	ENSMUSG00000070708.5
Sox8	1.27	2.40	0.03	0.34	1.79	4.30	ENSMUSG00000024176.10
Ifnb1	inf	2.40	0.00	0.00	0.00	1.40	ENSMUSG00000048806.4
Atp8a2	1.26	2.40	0.03	0.33	22.03	52.89	ENSMUSG00000021983.15
Iqcf1	1.26	2.40	0.01	0.24	117.66	282.39	ENSMUSG00000066383.6
Gata4	1.26	2.40	0.03	0.35	2.75	6.61	ENSMUSG00000021944.15
Fam71d	1.26	2.40	0.02	0.30	80.49	193.03	ENSMUSG00000056987.7
Atp1a4	1.26	2.40	0.02	0.30	83.57	200.39	ENSMUSG00000007107.6
Inpp4a	1.26	2.40	0.02	0.27	10.63	25.49	ENSMUSG00000026113.17
Scml2	1.26	2.40	0.02	0.32	3.72	8.90	ENSMUSG00000000037.16
Crtam	1.26	2.39	0.02	0.33	7.91	18.93	ENSMUSG00000032021.13
Txndc2	1.26	2.39	0.03	0.38	133.95	320.43	ENSMUSG00000050612.5
Hyal5	1.26	2.39	0.01	0.17	23.46	56.11	ENSMUSG00000029678.8
Eya4	1.26	2.39	0.03	0.35	10.17	24.31	ENSMUSG00000010461.14
Cdh2	1.26	2.39	0.01	0.21	8.38	20.02	ENSMUSG00000024304.14
Tmco2	1.26	2.39	0.01	0.20	144.00	343.94	ENSMUSG00000078577.3
Gzma	3.47	2.39	0.00	0.08	0.16	1.77	ENSMUSG00000023132.7
Csf2rb	2.70	2.38	0.02	0.26	0.34	2.19	ENSMUSG00000071713.4
Tex37	1.25	2.38	0.01	0.20	82.90	197.43	ENSMUSG00000051896.4
Cep128	1.25	2.38	0.04	0.40	51.61	122.80	ENSMUSG00000061533.15
Kcnk10	1.25	2.37	0.01	0.19	1.53	3.63	ENSMUSG00000033854.9

Spata19	1.25	2.37	0.02	0.32	226.76	537.65	ENSMUSG00000031991.9
Kif14	1.24	2.37	0.01	0.26	1.03	2.45	ENSMUSG00000041498.13
Erich3	1.24	2.37	0.04	0.44	71.70	169.94	ENSMUSG00000078161.8
Prss43	1.24	2.37	0.02	0.30	5.08	12.02	ENSMUSG00000058398.6
Bin2	1.24	2.36	0.02	0.31	7.82	18.49	ENSMUSG00000098112.7
1700084M14Rik	1.24	2.36	0.02	0.27	61.95	146.49	ENSMUSG00000057805.4
BC048507	1.24	2.36	0.02	0.29	39.81	94.12	ENSMUSG00000064063.5
1700093K21Rik	1.24	2.36	0.02	0.32	96.82	228.05	ENSMUSG00000020286.12
Asns	1.24	2.35	0.01	0.23	29.36	69.15	ENSMUSG00000029752.12
Vgll3	1.24	2.35	0.03	0.36	2.99	7.04	ENSMUSG00000091243.1
Wnt3	1.23	2.35	0.04	0.39	5.10	12.00	ENSMUSG00000000125.5
Rps6ka6	1.23	2.35	0.01	0.23	3.67	8.62	ENSMUSG00000025665.16
Tmsb15a	1.23	2.35	0.01	0.25	202.20	475.22	ENSMUSG00000060726.10
Frat2	1.23	2.35	0.02	0.27	15.37	36.10	ENSMUSG00000047604.2
Piwil1	1.23	2.35	0.01	0.17	48.70	114.30	ENSMUSG00000029423.10
Heatr9	1.23	2.34	0.03	0.37	6.90	16.17	ENSMUSG00000018925.3
Gm43786	1.23	2.34	0.03	0.37	53.25	124.85	ENSMUSG00000107252.1
Pdha2	1.23	2.34	0.01	0.21	35.51	83.13	ENSMUSG00000047674.2
Ccdc79	1.23	2.34	0.02	0.29	4.16	9.74	ENSMUSG00000052616.10
Ccdc182	1.23	2.34	0.02	0.32	10.82	25.31	ENSMUSG00000034031.5
Kif2b	1.23	2.34	0.03	0.34	148.90	348.17	ENSMUSG00000046755.5
Wbscr28	1.22	2.33	0.03	0.37	43.42	101.33	ENSMUSG00000040576.8
1700012B07Rik	1.22	2.33	0.02	0.27	82.51	192.52	ENSMUSG00000020617.13
Slc2a3	1.22	2.33	0.04	0.42	117.74	274.59	ENSMUSG00000003153.10
Sh3tc2	1.22	2.33	0.03	0.36	1.35	3.14	ENSMUSG00000045629.7
Fbn2	1.22	2.33	0.02	0.26	3.00	7.00	ENSMUSG00000024598.8
Ptpn20	1.22	2.33	0.01	0.22	13.13	30.55	ENSMUSG00000021940.9
H2afb1	1.22	2.32	0.01	0.18	160.52	372.99	ENSMUSG00000062651.4
Parvg	1.22	2.32	0.01	0.25	6.51	15.12	ENSMUSG00000022439.9
Tescl	1.22	2.32	0.02	0.28	73.33	170.32	ENSMUSG00000055826.5
Ankrd36	1.22	2.32	0.04	0.41	86.26	200.27	ENSMUSG00000020481.15
Gm4763	1.21	2.32	0.03	0.33	10.65	24.71	ENSMUSG00000058717.8
Hypm	1.21	2.32	0.03	0.37	85.48	198.39	ENSMUSG00000040456.2
Rtn1	1.21	2.32	0.03	0.33	7.71	17.89	ENSMUSG00000021087.17
Slc16a7	1.21	2.32	0.05	0.46	91.65	212.64	ENSMUSG00000020102.14
Trim45	1.21	2.32	0.01	0.25	9.09	21.08	ENSMUSG00000033233.17
1700034E13Rik	1.21	2.31	0.03	0.35	153.91	355.94	ENSMUSG00000024532.13
Fam222a	1.21	2.31	0.04	0.43	2.39	5.52	ENSMUSG00000041930.7
Gm17611	1.21	2.31	0.02	0.29	32.90	76.02	ENSMUSG00000090497.1
Proc	1.21	2.31	0.03	0.37	4.48	10.34	ENSMUSG00000024386.8
Paqr9	1.21	2.31	0.02	0.30	5.90	13.62	ENSMUSG00000064225.6
Shcbp1l	1.21	2.31	0.02	0.27	52.73	121.69	ENSMUSG00000042708.12
Gm9805	1.21	2.31	0.03	0.37	18.40	42.44	ENSMUSG00000046088.8
Nim1k	1.20	2.30	0.03	0.37	1.50	3.46	ENSMUSG00000095930.1
Zfp451	1.20	2.30	0.02	0.32	67.84	156.18	ENSMUSG00000042197.13
Aym1	1.20	2.30	0.04	0.43	3.19	7.35	ENSMUSG00000053873.1
Znrf4	1.20	2.30	0.03	0.37	140.59	323.15	ENSMUSG00000044526.2

Tdrd1	1.20	2.30	0.01	0.23	16.49	37.87	ENSMUSG00000025081.13
Sec1	1.20	2.30	0.04	0.44	8.86	20.33	ENSMUSG00000040364.7
Jazf1	1.20	2.29	0.01	0.23	12.88	29.56	ENSMUSG00000063568.11
AF366264	1.20	2.29	0.01	0.20	12.82	29.42	ENSMUSG00000057116.4
Bco1	1.20	2.29	0.03	0.35	6.17	14.15	ENSMUSG00000031845.15
Pwwp2b	1.20	2.29	0.02	0.32	78.38	179.76	ENSMUSG00000060260.12
Otub2	1.20	2.29	0.02	0.30	65.09	149.24	ENSMUSG00000021203.15
F10,F7	2.09	2.29	0.03	0.34	0.65	2.79	ENSMUSG00000031443.7
Izumo2	1.20	2.29	0.03	0.34	76.96	176.31	ENSMUSG00000066500.5
Meig1	1.19	2.29	0.03	0.34	1184.80	2710.77	ENSMUSG00000026650.15
Sufu	1.19	2.29	0.04	0.41	40.19	91.95	ENSMUSG00000025231.16
Spag4	1.19	2.29	0.05	0.45	101.20	231.52	ENSMUSG00000038180.11
Mak	1.19	2.29	0.04	0.39	22.92	52.42	ENSMUSG00000021363.13
Aif1	1.19	2.29	0.02	0.28	197.48	451.62	ENSMUSG00000024397.14
Mettl22	1.19	2.29	0.05	0.46	12.45	28.45	ENSMUSG00000039345.15
Galntl5	1.19	2.29	0.01	0.23	96.59	220.76	ENSMUSG00000028938.9
G6pd2	1.19	2.28	0.03	0.35	9.87	22.55	ENSMUSG00000089992.3
Clip4	1.19	2.28	0.03	0.35	90.42	206.56	ENSMUSG00000024059.9
Cylc2	1.19	2.28	0.03	0.34	52.80	120.62	ENSMUSG00000039555.13
1700017D01Rik	1.19	2.28	0.01	0.24	61.85	141.28	ENSMUSG00000024729.7
Prr19	1.19	2.28	0.02	0.30	19.84	45.27	ENSMUSG00000058741.4
Gm5941	1.19	2.28	0.03	0.37	86.80	198.02	ENSMUSG00000071726.2
2610318N02Rik	1.19	2.28	0.04	0.41	328.40	748.73	ENSMUSG00000049916.10
Spert	1.19	2.28	0.05	0.46	206.05	469.23	ENSMUSG00000034913.9
Arrdc5	1.19	2.28	0.02	0.32	64.69	147.30	ENSMUSG00000073380.1
Meioc	1.19	2.28	0.02	0.32	13.85	31.54	ENSMUSG00000051455.13
Scube2	1.19	2.27	0.03	0.34	4.48	10.19	ENSMUSG00000007279.14
Allc	1.18	2.27	0.04	0.42	118.40	269.11	ENSMUSG00000020636.14
Adam5	1.18	2.27	0.03	0.37	167.58	380.72	ENSMUSG00000031554.17
Zdhhc19	1.18	2.27	0.04	0.41	26.93	61.10	ENSMUSG00000052363.7
Zscan10	1.18	2.27	0.04	0.40	1.22	2.78	ENSMUSG00000023902.18
Prok2	1.18	2.27	0.03	0.33	19.03	43.13	ENSMUSG00000030069.15
Kif21b	1.18	2.27	0.01	0.23	4.83	10.94	ENSMUSG00000041642.18
Asb15	1.18	2.27	0.04	0.41	7.54	17.09	ENSMUSG00000029685.15
BC048671	1.18	2.27	0.04	0.43	53.80	121.87	ENSMUSG00000049694.13
Tex24	1.18	2.26	0.03	0.38	9.61	21.77	ENSMUSG00000071138.4
Iqcf3	1.18	2.26	0.03	0.34	269.19	609.36	ENSMUSG00000023577.14
Gm6729	1.18	2.26	0.02	0.31	6.85	15.51	ENSMUSG00000090816.1
Mlc1	1.18	2.26	0.02	0.32	8.42	19.03	ENSMUSG00000035805.13
Zfp382	1.18	2.26	0.03	0.35	3.81	8.62	ENSMUSG00000074220.10
Tex35	1.18	2.26	0.05	0.45	101.84	230.19	ENSMUSG00000026592.13
Fabp12	1.18	2.26	0.05	0.46	31.16	70.42	ENSMUSG00000027530.15
Ttc24	1.18	2.26	0.02	0.31	56.01	126.52	ENSMUSG00000051036.12
Ttc29	1.18	2.26	0.01	0.21	50.54	114.12	ENSMUSG00000037101.16
Topaz1	1.17	2.26	0.02	0.29	2.54	5.72	ENSMUSG00000094985.1
Enthd1	1.17	2.25	0.05	0.46	10.13	22.83	ENSMUSG00000050439.6
Map2	1.17	2.25	0.05	0.44	26.87	60.57	ENSMUSG00000015222.17

Irgc1	1.17	2.25	0.04	0.40	279.45	629.57	ENSMUSG00000062028.8
BC089491	1.17	2.25	0.02	0.32	86.96	195.84	ENSMUSG00000046750.17
Gm8369, Ms4a4b, Ms4a6c	1.17	2.25	0.03	0.37	2.75	6.18	ENSMUSG00000079419.4
Spta1	1.17	2.25	0.04	0.40	3.10	6.98	ENSMUSG00000026532.7
Ropn1	1.17	2.25	0.03	0.37	193.37	435.32	ENSMUSG00000022832.10
Armc12	1.17	2.25	0.01	0.23	85.08	191.41	ENSMUSG00000024223.2
Mgat5	1.17	2.25	0.02	0.31	6.73	15.14	ENSMUSG00000036155.13
Cdc42ep3	1.17	2.25	0.02	0.32	31.07	69.89	ENSMUSG00000036533.8
Cep85l	1.17	2.25	0.05	0.46	32.17	72.32	ENSMUSG00000038594.8
Wdr20rt	1.17	2.25	0.03	0.34	24.49	55.07	ENSMUSG00000035560.4
Gm7356	1.17	2.25	0.05	0.45	7.52	16.89	ENSMUSG00000101307.1
Tmco5	1.17	2.25	0.02	0.30	124.41	279.37	ENSMUSG00000027355.15
Rp1	1.17	2.25	0.03	0.36	2.61	5.85	ENSMUSG00000025900.11
Kif2c	1.17	2.25	0.01	0.25	60.07	134.87	ENSMUSG00000028678.13
Cfap206	1.17	2.24	0.02	0.32	154.79	347.34	ENSMUSG00000028294.15
Tbc1d21	1.17	2.24	0.03	0.39	41.23	92.50	ENSMUSG00000036244.4
Syce3	1.17	2.24	0.02	0.31	80.44	180.42	ENSMUSG00000078938.9
BC051628	1.17	2.24	0.04	0.42	80.58	180.69	ENSMUSG00000047841.8
1700029P11Rik	1.16	2.24	0.01	0.23	43.77	98.14	ENSMUSG00000061633.3
St8sia3	1.16	2.24	0.04	0.40	9.43	21.13	ENSMUSG00000056812.13
Zbtb3	1.16	2.24	0.02	0.33	10.57	23.66	ENSMUSG00000071661.6
1700013G24Rik	1.16	2.24	0.02	0.29	256.66	574.37	ENSMUSG00000041399.3
Sycp2	1.16	2.24	0.03	0.35	21.29	47.65	ENSMUSG00000060445.11
Tex26	1.16	2.24	0.02	0.30	36.29	81.19	ENSMUSG00000029660.10
Abcc12	1.16	2.24	0.02	0.30	15.57	34.84	ENSMUSG00000036872.16
Them6	1.16	2.24	0.02	0.27	13.68	30.60	ENSMUSG00000056665.2
Dkkl1	1.16	2.24	0.04	0.39	458.12	1024.05	ENSMUSG00000030792.7
Csnka2ip	1.16	2.23	0.02	0.27	55.73	124.49	ENSMUSG00000068167.4
Uba1y	1.16	2.23	0.04	0.42	1.75	3.92	ENSMUSG00000069053.11
Tcam1	1.16	2.23	0.02	0.28	24.33	54.31	ENSMUSG00000020712.11
Sphkap	1.16	2.23	0.02	0.29	9.57	21.36	ENSMUSG00000026163.17
Ctag2	1.16	2.23	0.02	0.33	33.09	73.82	ENSMUSG00000031181.2
Ccer1	1.16	2.23	0.02	0.28	111.42	248.53	ENSMUSG00000047025.4
Aard	1.16	2.23	0.01	0.25	31.20	69.54	ENSMUSG00000068522.4
Spata21	1.16	2.23	0.04	0.43	193.94	432.27	ENSMUSG00000045004.3
4930590J08Rik	1.16	2.23	0.03	0.36	25.76	57.38	ENSMUSG00000034063.8
Ankef1	1.16	2.23	0.02	0.26	89.49	199.37	ENSMUSG00000074771.10
Cdc7	1.16	2.23	0.02	0.31	3.26	7.26	ENSMUSG00000029283.17
4933402J07Rik	1.16	2.23	0.02	0.28	118.49	263.88	ENSMUSG00000069971.5
4933436I01Rik	1.15	2.23	0.03	0.34	9.25	20.60	ENSMUSG00000025288.1
1700011E24Rik	1.15	2.23	0.02	0.28	160.29	356.79	ENSMUSG00000036557.8
Oplah	1.15	2.23	0.05	0.46	14.92	33.21	ENSMUSG00000022562.13
Ccdc28a	1.15	2.23	0.04	0.44	26.29	58.52	ENSMUSG00000059554.12
Poc1a	1.15	2.23	0.04	0.40	14.92	33.20	ENSMUSG00000023345.15
Muc1	1.15	2.23	0.03	0.35	5.13	11.42	ENSMUSG00000042784.9
Lyrm1	1.15	2.22	0.04	0.43	16.50	36.70	ENSMUSG00000030922.12
Cdrt4	1.15	2.22	0.02	0.31	159.24	354.19	ENSMUSG00000042200.3

Pde10a	1.15	2.22	0.05	0.46	5.77	12.84	ENSMUSG00000023868.15
Bhmt	1.15	2.22	0.02	0.30	8.30	18.46	ENSMUSG00000074768.5
Gm5142	1.15	2.22	0.01	0.25	111.83	248.71	ENSMUSG00000053868.3
Actl9	1.15	2.22	0.02	0.29	125.59	279.22	ENSMUSG00000092519.2
Adcy10	1.15	2.22	0.01	0.22	34.57	76.83	ENSMUSG00000026567.16
4930505A04Rik	1.15	2.22	0.03	0.35	63.13	140.29	ENSMUSG00000040919.13
Gstt4	1.15	2.22	0.02	0.28	103.12	229.07	ENSMUSG00000009093.7
Kap	1.15	2.22	0.01	0.22	51.41	114.16	ENSMUSG00000032758.4
Sncaip	1.15	2.22	0.01	0.20	7.07	15.69	ENSMUSG00000024534.15
Myo1a	1.15	2.22	0.04	0.39	2.84	6.31	ENSMUSG00000025401.8
Stat1, Stat4	1.15	2.22	0.04	0.41	77.30	171.49	ENSMUSG00000026104.14
Calr3, Cherp	1.15	2.22	0.04	0.39	45.59	101.09	ENSMUSG00000052488.6
Slc8a2	1.15	2.22	0.02	0.31	7.45	16.51	ENSMUSG00000030376.7
Slc25a41	1.15	2.22	0.04	0.40	7.72	17.11	ENSMUSG00000011486.14
1700031M16Rik	1.15	2.21	0.04	0.40	109.56	242.63	ENSMUSG00000099353.6
Mybl1	1.15	2.21	0.01	0.19	7.17	15.86	ENSMUSG00000025912.16
Lrrc27	1.15	2.21	0.03	0.34	135.05	298.88	ENSMUSG00000015980.14
4921506M07Rik	1.15	2.21	0.04	0.40	13.85	30.65	ENSMUSG00000046782.14
Rnf32	1.15	2.21	0.02	0.26	89.14	197.26	ENSMUSG00000029130.12
Cmtm1, Cmtm2a	1.15	2.21	0.02	0.29	260.42	576.18	ENSMUSG00000031876.16
Cd109	1.15	2.21	0.02	0.32	4.21	9.31	ENSMUSG00000046186.8
Spata20	1.14	2.21	0.04	0.41	223.98	495.18	ENSMUSG00000020867.13
Pla2g10	1.14	2.21	0.03	0.37	13.79	30.47	ENSMUSG00000022683.13
Ranbp17	1.14	2.21	0.03	0.38	11.57	25.57	ENSMUSG00000040594.19
Gm6760	1.14	2.21	0.03	0.36	105.79	233.63	ENSMUSG00000079579.3
Ankar	1.14	2.21	0.04	0.42	11.33	24.97	ENSMUSG00000039342.4
Cfap46	1.14	2.20	0.05	0.44	10.81	23.84	ENSMUSG00000049571.16
Efcab9	1.14	2.20	0.02	0.28	63.94	140.94	ENSMUSG00000044056.3
1700011L22Rik	1.14	2.20	0.02	0.28	68.96	151.98	ENSMUSG00000031682.4
Spatc1	1.14	2.20	0.03	0.37	57.63	127.00	ENSMUSG00000049653.4
Lrrfip2	1.14	2.20	0.01	0.25	85.30	187.86	ENSMUSG00000032497.15
Lyzl1	1.14	2.20	0.02	0.30	44.92	98.86	ENSMUSG00000024233.10
Zcchc13	1.14	2.20	0.02	0.27	22.83	50.23	ENSMUSG00000031330.8
Hdhd1a	1.14	2.20	0.02	0.32	31.62	69.55	ENSMUSG00000048875.2
Efhc2	1.14	2.20	0.03	0.36	5.62	12.36	ENSMUSG00000025038.7
Prps1l1	1.14	2.20	0.01	0.25	58.47	128.52	ENSMUSG00000092305.1
Kif19a	1.14	2.20	0.03	0.39	3.85	8.46	ENSMUSG00000010021.13
1700024P04Rik	1.13	2.20	0.02	0.30	208.29	457.43	ENSMUSG00000045022.6
Cxcl13	3.37	2.19	0.02	0.30	0.15	1.52	ENSMUSG00000023078.6
Mroh2b	1.13	2.19	0.03	0.33	63.08	138.44	ENSMUSG00000022155.8
Ehbp1l1	1.13	2.19	0.05	0.47	16.80	36.85	ENSMUSG00000024937.14
Csf3r	2.79	2.19	0.00	0.01	0.25	1.75	ENSMUSG00000028859.14
Mael	1.13	2.19	0.02	0.32	280.77	615.82	ENSMUSG00000040629.8
Tnfaip2	1.13	2.19	0.01	0.23	20.41	44.75	ENSMUSG00000021281.15
1700013D24Rik	1.13	2.19	0.02	0.32	89.96	197.22	ENSMUSG00000079346.3
Gm14569	1.13	2.19	0.02	0.32	10.16	22.28	ENSMUSG00000091556.8
Tmem95	1.13	2.19	0.04	0.42	2.95	6.45	ENSMUSG00000094845.1

Nipsnap3a	1.13	2.19	0.04	0.39	30.15	66.04	ENSMUSG00000015242.14
Arhgef6	1.13	2.19	0.04	0.42	9.99	21.88	ENSMUSG00000031133.12
Cdk5rap2	1.13	2.19	0.02	0.32	35.20	77.00	ENSMUSG00000039298.16
Rnf19b	1.13	2.19	0.04	0.40	120.14	262.79	ENSMUSG00000028793.15
Cdc14a	1.13	2.19	0.02	0.32	35.32	77.23	ENSMUSG00000033502.14
4930522H14Rik	1.13	2.19	0.02	0.30	23.51	51.41	ENSMUSG00000060491.14
Gfra2	1.13	2.19	0.05	0.47	9.35	20.45	ENSMUSG00000022103.9
Odf2	1.13	2.19	0.03	0.37	995.73	2175.82	ENSMUSG00000026790.19
Mov10l1	1.13	2.18	0.02	0.31	13.25	28.94	ENSMUSG00000015365.15
D1Pas1	1.13	2.18	0.02	0.29	47.36	103.42	ENSMUSG00000039224.1
Capn11	1.13	2.18	0.02	0.31	9.49	20.71	ENSMUSG00000058626.16
Mroh4	1.13	2.18	0.02	0.30	91.18	199.00	ENSMUSG00000022603.9
Ccdc89	1.13	2.18	0.02	0.32	42.73	93.26	ENSMUSG00000044362.8
Tbata	1.12	2.18	0.02	0.31	86.87	189.47	ENSMUSG00000020096.19
Trim36	1.12	2.18	0.02	0.26	60.38	131.66	ENSMUSG00000033949.11
Fscb	1.12	2.18	0.03	0.34	36.49	79.56	ENSMUSG00000043060.7
Pifo	1.12	2.18	0.04	0.40	46.53	101.42	ENSMUSG00000010136.14
Prdx6b	1.12	2.18	0.04	0.40	66.96	145.94	ENSMUSG00000050114.7
Pmfbp1	1.12	2.18	0.04	0.41	83.99	183.04	ENSMUSG00000031727.7
Ankrd53	1.12	2.18	0.03	0.37	16.97	36.97	ENSMUSG00000014747.6
1700034O15Rik	1.12	2.18	0.03	0.37	191.27	416.73	ENSMUSG00000029867.5
Letm2	1.12	2.18	0.04	0.44	48.12	104.83	ENSMUSG00000037363.8
Slc22a16	1.12	2.18	0.02	0.30	42.60	92.77	ENSMUSG00000019834.15
Serpina3a	1.12	2.18	0.04	0.43	5.91	12.87	ENSMUSG00000041536.13
Cdc34	1.12	2.18	0.03	0.34	130.23	283.55	ENSMUSG00000020307.14
E2f8	1.12	2.18	0.04	0.43	1.86	4.04	ENSMUSG00000046179.17
Chl1	1.12	2.17	0.02	0.32	106.34	231.22	ENSMUSG00000030077.11
Prkcq	1.12	2.17	0.01	0.24	33.31	72.39	ENSMUSG00000026778.13
Prr30	1.12	2.17	0.04	0.41	112.59	244.56	ENSMUSG00000042888.10
Adam24	1.12	2.17	0.02	0.29	16.24	35.28	ENSMUSG00000046723.3
Reep2	1.12	2.17	0.02	0.32	24.04	52.18	ENSMUSG00000038555.7
Knstrn	1.12	2.17	0.05	0.45	50.88	110.43	ENSMUSG00000027331.15
Ccdc67	1.12	2.17	0.04	0.40	51.57	111.90	ENSMUSG00000039977.16
Efcab5	1.12	2.17	0.02	0.28	30.93	67.10	ENSMUSG00000050944.14
Klk1b8	1.12	2.17	0.04	0.42	58.60	127.09	ENSMUSG00000063089.4
Tppp2	1.12	2.17	0.03	0.33	175.20	379.88	ENSMUSG00000008813.12
1700009N14Rik	1.12	2.17	0.03	0.37	201.02	435.76	ENSMUSG00000028287.4
1700029H14Rik	1.12	2.17	0.03	0.35	190.39	412.72	ENSMUSG00000031452.14
4922502D21Rik	1.12	2.17	0.04	0.41	267.78	580.42	ENSMUSG00000047720.7
Cdkl5	1.12	2.17	0.02	0.32	4.29	9.31	ENSMUSG00000031292.14
Ccp110	1.12	2.17	0.02	0.31	23.04	49.93	ENSMUSG00000033904.16
Pdcl2	1.12	2.17	0.02	0.32	179.58	389.11	ENSMUSG00000029235.14
Cetn1	1.12	2.17	0.03	0.34	152.72	330.87	ENSMUSG00000050996.6
Odf4	1.11	2.17	0.05	0.44	75.72	164.00	ENSMUSG00000032921.13
Fbp1	1.11	2.17	0.02	0.31	150.52	325.97	ENSMUSG00000069805.10
Rpl39l	1.11	2.17	0.03	0.33	142.88	309.34	ENSMUSG00000039209.12
Ubqln3	1.11	2.16	0.02	0.32	85.62	185.35	ENSMUSG00000051618.5

Fxyd5,Fxyd7	1.11	2.16	0.04	0.41	11.72	25.38	ENSMUSG00000009687.14
Saysd1	1.11	2.16	0.01	0.24	48.85	105.70	ENSMUSG00000045107.4
Tgfbi	1.11	2.16	0.01	0.16	10.43	22.55	ENSMUSG00000035493.9
Nek2	1.11	2.16	0.02	0.33	34.81	75.26	ENSMUSG00000026622.15
Efhc1	1.11	2.16	0.03	0.35	78.48	169.62	ENSMUSG00000041809.5
Ppp3r2	1.11	2.16	0.03	0.36	63.14	136.41	ENSMUSG00000028310.2
Syce1	1.11	2.16	0.02	0.32	99.53	214.94	ENSMUSG00000025480.4
Akap1	1.11	2.16	0.05	0.46	114.28	246.78	ENSMUSG00000018428.15
Adam3	1.11	2.16	0.05	0.44	289.49	625.09	ENSMUSG00000031553.15
1700012A03Rik	1.11	2.16	0.02	0.28	105.48	227.71	ENSMUSG00000029766.7
Stk33	1.11	2.16	0.04	0.40	59.73	128.91	ENSMUSG00000031027.15
4930524B15Rik	1.11	2.16	0.02	0.32	9.36	20.20	ENSMUSG00000020299.6
Papolb, Radil	1.11	2.16	0.03	0.35	72.78	157.02	ENSMUSG00000029576.17
Nckap1l	1.11	2.16	0.02	0.29	11.78	25.41	ENSMUSG00000022488.8
Catsperd	1.11	2.16	0.05	0.46	20.86	45.00	ENSMUSG00000040828.9
Atoh8	1.11	2.16	0.03	0.38	43.37	93.52	ENSMUSG00000037621.8
Sohlh2	1.11	2.15	0.05	0.46	1.87	4.02	ENSMUSG00000027794.4
Pabpc6	1.11	2.15	0.03	0.34	60.83	131.01	ENSMUSG00000046173.2
Bbs9	1.11	2.15	0.03	0.37	11.64	25.08	ENSMUSG00000035919.16
Fam170b	1.11	2.15	0.05	0.46	55.56	119.63	ENSMUSG00000078127.2
CK137956	1.11	2.15	0.03	0.34	28.56	61.48	ENSMUSG00000028813.2
Hsfy2	1.11	2.15	0.04	0.40	31.23	67.21	ENSMUSG00000045336.5
Tpbgl	1.11	2.15	0.05	0.45	2.50	5.38	ENSMUSG00000096606.2
Gtsf1	1.11	2.15	0.04	0.41	143.03	307.80	ENSMUSG00000022487.14
1700003H04Rik	1.11	2.15	0.02	0.28	61.17	131.59	ENSMUSG00000039174.14
Spata16	1.11	2.15	0.03	0.34	72.82	156.66	ENSMUSG00000039335.11
Gtf2a1l	1.11	2.15	0.02	0.30	72.71	156.42	ENSMUSG00000024154.10
Rfx2	1.10	2.15	0.03	0.35	54.50	117.21	ENSMUSG00000024206.14
Akr1cl	1.10	2.15	0.02	0.27	22.52	48.42	ENSMUSG00000025955.13
Afap1	1.10	2.15	0.03	0.38	10.58	22.75	ENSMUSG00000029094.11
Gm884	1.10	2.15	0.05	0.46	13.26	28.50	ENSMUSG00000034239.14
Ttk	1.10	2.15	0.02	0.28	9.16	19.68	ENSMUSG00000038379.15
Spaca1	1.10	2.15	0.02	0.31	62.03	133.31	ENSMUSG00000028264.16
BC051142	1.10	2.15	0.02	0.26	186.59	400.92	ENSMUSG00000057246.14
Zbbx	1.10	2.15	0.03	0.36	61.23	131.55	ENSMUSG00000034151.13
Cabs1	1.10	2.15	0.03	0.36	161.24	346.29	ENSMUSG00000007907.4
Ahi1	1.10	2.15	0.02	0.26	21.44	46.04	ENSMUSG00000019986.16
Rab11fip5	1.10	2.15	0.04	0.40	36.72	78.85	ENSMUSG00000051343.11
Ube2u	1.10	2.15	0.01	0.26	27.83	59.74	ENSMUSG00000069733.11
4933415F23Rik	1.10	2.15	0.02	0.27	93.97	201.72	ENSMUSG00000073730.2
Mlf1	1.10	2.15	0.02	0.29	216.66	465.08	ENSMUSG00000048416.15
Dpep3	1.10	2.15	0.04	0.39	77.69	166.76	ENSMUSG00000031898.8
Ipo5	1.10	2.15	0.05	0.46	125.25	268.82	ENSMUSG00000030662.9
Armc3	1.10	2.15	0.04	0.41	30.41	65.23	ENSMUSG00000037683.14
4933402E13Rik	1.10	2.14	0.03	0.39	6.47	13.86	ENSMUSG00000045330.8
AA467197	1.10	2.14	0.03	0.37	96.94	207.79	ENSMUSG00000033213.16
Hrasls5	1.10	2.14	0.04	0.43	416.41	892.52	ENSMUSG00000024973.16

Corin	1.10	2.14	0.05	0.47	3.32	7.10	ENSMUSG00000005220.10
Spaca7	1.10	2.14	0.02	0.27	64.38	137.93	ENSMUSG00000010435.6
Gp1bb, Sept5	1.10	2.14	0.02	0.31	17.95	38.45	ENSMUSG00000072214.6
Nuf2	1.10	2.14	0.01	0.23	15.66	33.54	ENSMUSG00000026683.14
Actl11, Camkv, Traip	1.10	2.14	0.02	0.29	58.06	124.34	ENSMUSG00000032586.9
Tex13a	1.10	2.14	0.02	0.32	17.65	37.81	ENSMUSG00000071686.2
Lelp1	1.10	2.14	0.03	0.36	151.05	323.48	ENSMUSG00000104791.1
Tcp10a	1.10	2.14	0.04	0.40	75.63	161.91	ENSMUSG00000071322.12
Rb1cc1	1.10	2.14	0.04	0.40	72.81	155.86	ENSMUSG00000025907.14
Lyz2	1.10	2.14	0.01	0.18	28.50	60.96	ENSMUSG00000069516.7
Gykl1	1.10	2.14	0.02	0.31	101.22	216.49	ENSMUSG00000053624.3
Thop1	1.10	2.14	0.03	0.36	63.74	136.30	ENSMUSG00000004929.12
Alms1	1.10	2.14	0.02	0.27	9.41	20.10	ENSMUSG00000063810.6
Rasa3	1.09	2.14	0.02	0.29	12.74	27.22	ENSMUSG00000031453.15
Slco6c1	1.09	2.13	0.03	0.35	20.63	44.03	ENSMUSG00000026331.13
Crisp2	1.09	2.13	0.04	0.40	599.98	1280.28	ENSMUSG00000023930.13
Stil	1.09	2.13	0.01	0.25	3.55	7.57	ENSMUSG00000028718.16
Lgals3	1.09	2.13	0.02	0.33	10.24	21.85	ENSMUSG00000050335.16
Spata31	1.09	2.13	0.02	0.29	31.74	67.70	ENSMUSG00000056223.7
Cenpv	1.09	2.13	0.02	0.28	141.20	301.11	ENSMUSG00000018509.8
Ubqlnl	1.09	2.13	0.04	0.39	60.52	129.06	ENSMUSG00000051437.4
Crem	1.09	2.13	0.04	0.40	94.92	202.33	ENSMUSG00000063889.16
Ace3	1.09	2.13	0.04	0.40	16.86	35.94	ENSMUSG00000101605.1
4933416C03Rik	1.09	2.13	0.04	0.44	37.06	79.00	ENSMUSG00000074734.2
Zfp217	1.09	2.13	0.03	0.35	25.12	53.52	ENSMUSG00000052056.14
Cpa5	1.09	2.13	0.05	0.45	16.31	34.75	ENSMUSG00000029788.13
Bpifa3	1.09	2.13	0.03	0.35	32.00	68.15	ENSMUSG00000027482.12
Gm498	1.09	2.13	0.03	0.35	43.34	92.25	ENSMUSG00000031085.16
Cep57l1	1.09	2.13	0.02	0.29	15.45	32.89	ENSMUSG00000019813.15
Zfp474	1.09	2.13	0.03	0.37	27.69	58.93	ENSMUSG00000046886.5
Cmtm2b	1.09	2.13	0.03	0.34	205.00	436.31	ENSMUSG00000035785.5
Adam2	1.09	2.13	0.02	0.33	59.82	127.29	ENSMUSG00000022039.5
Bnipl, Gm128	1.09	2.13	0.03	0.39	111.02	236.22	ENSMUSG00000028115.16
Osgin1	1.09	2.13	0.04	0.43	55.36	117.73	ENSMUSG00000074063.9
1700010I14Rik	1.09	2.13	0.03	0.34	131.44	279.33	ENSMUSG00000023873.12
Cox7b2	1.09	2.13	0.02	0.31	114.65	243.64	ENSMUSG00000049387.9
Catsperg2	1.09	2.12	0.04	0.40	18.36	39.01	ENSMUSG00000049123.10
Iqcf4	1.09	2.12	0.02	0.27	132.58	281.62	ENSMUSG00000041009.8
Lyrm7	1.09	2.12	0.02	0.29	14.01	29.74	ENSMUSG00000020268.13
Pi4k2b	1.09	2.12	0.01	0.23	18.98	40.31	ENSMUSG00000029186.12
Fn1	1.09	2.12	0.02	0.33	5.25	11.14	ENSMUSG00000026193.15
Gm1141	1.09	2.12	0.03	0.38	13.85	29.39	ENSMUSG00000073130.8
Csl	1.08	2.12	0.03	0.35	50.73	107.57	ENSMUSG00000046934.4
Scmh1, Slfnl1	1.08	2.12	0.05	0.44	119.88	254.17	ENSMUSG00000000085.16
Tmem247	1.08	2.12	0.03	0.39	143.44	304.02	ENSMUSG00000037689.6
Wfdc15a	1.08	2.12	0.02	0.32	72.19	152.97	ENSMUSG00000051769.2
Fsip2	1.08	2.12	0.02	0.31	12.56	26.62	ENSMUSG00000075249.12

Ppm1f	1.08	2.12	0.03	0.37	5.69	12.06	ENSMUSG00000026181.11
Aldoart2	1.08	2.12	0.03	0.33	107.30	227.15	ENSMUSG00000063129.1
Slc9c1	1.08	2.12	0.03	0.38	14.96	31.66	ENSMUSG00000033210.16
Kif1a	1.08	2.12	0.02	0.28	2.48	5.24	ENSMUSG00000014602.15
Nol4	1.08	2.12	0.03	0.38	16.66	35.24	ENSMUSG00000041923.15
Adam20, Adam25, Adam39	1.08	2.11	0.04	0.42	25.12	53.11	ENSMUSG00000054033.5
Ccdc13	1.08	2.11	0.02	0.33	28.58	60.42	ENSMUSG00000079235.10
Spag6l	1.08	2.11	0.02	0.28	62.70	132.51	ENSMUSG00000022783.11
Ccdc150	1.08	2.11	0.02	0.27	26.15	55.22	ENSMUSG00000025983.11
4930504O13Rik	1.08	2.11	0.03	0.37	63.71	134.52	ENSMUSG00000052642.3
Gm6812	1.08	2.11	0.04	0.40	26.14	55.19	ENSMUSG00000056815.1
Aldoart1	1.08	2.11	0.03	0.38	31.16	65.78	ENSMUSG00000059343.4
Ubl4b	1.08	2.11	0.04	0.39	91.72	193.64	ENSMUSG00000055891.7
Nme8	1.08	2.11	0.02	0.32	34.35	72.52	ENSMUSG00000041138.15
Ppef1	1.08	2.11	0.05	0.46	4.17	8.80	ENSMUSG00000062168.12
BC030867	1.08	2.11	0.05	0.46	4.19	8.83	ENSMUSG00000034773.16
Kbtbd8	1.08	2.11	0.05	0.45	1.79	3.77	ENSMUSG00000030031.14
Cyct	1.08	2.11	0.02	0.27	57.12	120.50	ENSMUSG00000056436.4
Pik3cd	1.08	2.11	0.03	0.36	6.76	14.26	ENSMUSG00000039936.18
Pim1	1.08	2.11	0.02	0.32	16.95	35.74	ENSMUSG00000024014.7
1700001L19Rik	1.08	2.11	0.02	0.33	27.15	57.23	ENSMUSG00000021534.7
Txndc8	1.08	2.11	0.04	0.44	102.04	215.03	ENSMUSG00000038709.14
Plekho1	1.07	2.11	0.04	0.43	15.78	33.24	ENSMUSG00000015745.9
Pxt1	1.07	2.11	0.04	0.43	33.18	69.85	ENSMUSG00000045378.4
Fam186b	1.07	2.11	0.02	0.33	19.21	40.44	ENSMUSG00000078907.1
1700016K19Rik	1.07	2.10	0.03	0.38	95.09	200.17	ENSMUSG00000053783.5
Fam50b	1.07	2.10	0.05	0.47	82.15	172.88	ENSMUSG00000038246.6
1700029F12Rik	1.07	2.10	0.05	0.45	255.83	538.34	ENSMUSG00000052075.6
Pom121l2	1.07	2.10	0.03	0.36	34.98	73.60	ENSMUSG00000016982.6
Lipe	1.07	2.10	0.04	0.40	66.55	140.00	ENSMUSG00000003123.15
Mesp1	1.07	2.10	0.02	0.31	29.37	61.79	ENSMUSG00000030544.5
Myl10	1.07	2.10	0.04	0.42	64.10	134.84	ENSMUSG00000005474.9
Pgk2	1.07	2.10	0.03	0.38	200.87	422.41	ENSMUSG00000031233.5
Zfp821	1.07	2.10	0.03	0.38	22.94	48.24	ENSMUSG00000031728.9
Epb41l2	1.07	2.10	0.02	0.28	40.14	84.39	ENSMUSG00000019978.15
Rfx8	1.07	2.10	0.02	0.31	14.33	30.12	ENSMUSG00000057173.8
Zdhhc25	1.07	2.10	0.05	0.46	23.86	50.14	ENSMUSG00000054117.8
Gm9999	1.07	2.10	0.04	0.43	759.63	1596.43	ENSMUSG00000056509.10
Il3ra	1.07	2.10	0.04	0.44	11.20	23.51	ENSMUSG00000068758.7
Setx	1.07	2.10	0.04	0.40	66.13	138.87	ENSMUSG00000043535.13
Cst9	1.07	2.10	0.03	0.39	80.95	169.97	ENSMUSG00000027445.3
1700021F07Rik	1.07	2.10	0.02	0.28	103.33	216.96	ENSMUSG00000027518.3
Ttc39d	1.07	2.10	0.03	0.34	55.83	117.19	ENSMUSG00000046196.4
Vsig1	1.07	2.10	0.04	0.39	33.14	69.54	ENSMUSG00000031430.8
Dtx1	1.07	2.10	0.03	0.35	4.09	8.58	ENSMUSG00000029603.15
Abhd5	1.07	2.10	0.04	0.40	53.32	111.84	ENSMUSG00000032540.15
Nol8	1.07	2.10	0.02	0.30	28.85	60.50	ENSMUSG00000021392.7

Dsg1a	1.07	2.09	0.03	0.36	3.46	7.25	ENSMUSG00000069441.3
Cfap221	1.07	2.09	0.04	0.41	6.97	14.60	ENSMUSG00000036962.12
Tex101	1.07	2.09	0.03	0.34	67.52	141.35	ENSMUSG00000062773.6
Parvb	1.07	2.09	0.03	0.38	1.54	3.22	ENSMUSG00000022438.6
Acrv1	1.07	2.09	0.05	0.44	77.79	162.80	ENSMUSG00000032110.4
Hipk4	1.07	2.09	0.04	0.40	24.25	50.74	ENSMUSG00000040424.15
Capn6	1.06	2.09	0.04	0.44	1.51	3.15	ENSMUSG00000067276.5
Nfatc2ip	1.06	2.09	0.02	0.26	10.69	22.33	ENSMUSG00000030722.7
Paqr8	1.06	2.09	0.04	0.44	5.45	11.39	ENSMUSG00000025931.15
Ccdc181	1.06	2.09	0.03	0.35	96.83	202.24	ENSMUSG00000026578.6
Morc2b	1.06	2.09	0.03	0.38	42.82	89.40	ENSMUSG00000048602.8
Satl1	1.06	2.09	0.04	0.41	10.19	21.27	ENSMUSG00000025527.9
Socs3	2.01	2.09	0.05	0.46	0.56	2.25	ENSMUSG00000053113.3
Ankrd9	1.06	2.09	0.03	0.34	19.77	41.27	ENSMUSG00000037904.14
Pou2f2	1.06	2.09	0.03	0.34	2.11	4.41	ENSMUSG00000008496.18
Rusc2	1.06	2.09	0.03	0.37	9.73	20.30	ENSMUSG00000035969.15
Ubxn10	1.06	2.09	0.04	0.42	55.49	115.72	ENSMUSG00000043621.13
Rwdd2a	1.06	2.08	0.05	0.44	18.25	38.05	ENSMUSG00000032417.10
Odf1	1.06	2.08	0.04	0.43	538.07	1121.72	ENSMUSG00000061923.4
4933405O20Rik	1.06	2.08	0.05	0.46	21.28	44.36	ENSMUSG00000084234.2
Klf4	1.06	2.08	0.02	0.30	32.16	67.03	ENSMUSG00000003032.8
Fads6, Fdxr	1.06	2.08	0.04	0.41	7.70	16.04	ENSMUSG00000018861.8
Rpl10l	1.06	2.08	0.02	0.30	75.90	158.12	ENSMUSG00000060499.7
Hip1, Pom121	1.06	2.08	0.02	0.31	27.43	57.10	ENSMUSG00000039959.12
Cfap70	1.06	2.08	0.05	0.45	28.58	59.48	ENSMUSG00000039543.15
Pebp4	1.06	2.08	0.03	0.35	96.16	200.10	ENSMUSG00000022085.3
Fabp9, Gm37389, Pmp2	1.06	2.08	0.04	0.44	1010.30	2101.76	ENSMUSG00000027528.12
Lrrc8b	1.06	2.08	0.05	0.45	52.00	108.13	ENSMUSG00000070639.5
Dnah12	1.06	2.08	0.05	0.45	8.94	18.57	ENSMUSG00000021879.12
Slco6d1	1.06	2.08	0.05	0.46	10.72	22.28	ENSMUSG00000026336.13
Pvrl3	1.06	2.08	0.05	0.46	72.55	150.74	ENSMUSG00000022656.15
BB014433	1.05	2.08	0.04	0.41	107.01	222.25	ENSMUSG00000049008.4
BC100451, Timp2	1.05	2.08	0.04	0.43	167.99	348.87	ENSMUSG00000076433.4
Arl14epl	1.05	2.08	0.05	0.46	6.21	12.90	ENSMUSG00000073568.1
4930453N24Rik	1.05	2.08	0.04	0.44	133.55	277.17	ENSMUSG00000059920.9
D7Ertd443e	1.05	2.08	0.04	0.41	26.15	54.26	ENSMUSG00000108569.1
Efhd1	1.05	2.07	0.04	0.41	83.47	173.20	ENSMUSG00000026255.15
1700025B11Rik	1.05	2.07	0.04	0.40	31.53	65.41	ENSMUSG00000101113.1
Fkbpl	1.05	2.07	0.04	0.39	67.54	140.05	ENSMUSG00000033739.8
Ccdc81	1.05	2.07	0.03	0.34	49.20	101.88	ENSMUSG00000039391.11
Fam170a	1.05	2.07	0.04	0.41	28.02	58.01	ENSMUSG00000035420.6
Rnf44	1.05	2.07	0.03	0.39	54.01	111.81	ENSMUSG00000034928.16
Srpk1	1.05	2.07	0.05	0.44	88.14	182.48	ENSMUSG00000004865.15
Rbm44	1.05	2.07	0.02	0.32	14.04	29.06	ENSMUSG00000070732.2
Nphp1	1.05	2.07	0.04	0.43	169.87	351.56	ENSMUSG00000027378.16
Spaca3	1.05	2.07	0.03	0.38	83.67	173.15	ENSMUSG00000053184.15
Piwil2	1.05	2.07	0.02	0.33	16.17	33.45	ENSMUSG00000033644.4

Ehd1	1.05	2.07	0.02	0.32	73.93	152.91	ENSMUSG00000024772.9
Ankrd60	1.05	2.07	0.04	0.39	75.34	155.80	ENSMUSG00000027517.13
Dazl	1.05	2.07	0.02	0.27	15.72	32.50	ENSMUSG00000010592.8
Lrrcc1	1.05	2.07	0.03	0.38	38.09	78.67	ENSMUSG00000027550.14
Bcl2l14	1.05	2.07	0.03	0.37	98.76	203.99	ENSMUSG00000030200.13
Fam110a	1.05	2.06	0.02	0.30	39.37	81.29	ENSMUSG00000027459.16
Ddhd1	1.05	2.06	0.04	0.43	56.36	116.35	ENSMUSG00000037697.18
Tekt1	1.05	2.06	0.04	0.41	122.00	251.84	ENSMUSG00000020799.16
Cyth1	1.05	2.06	0.04	0.40	14.75	30.45	ENSMUSG00000017132.17
Slc25a31	1.04	2.06	0.03	0.36	9.44	19.49	ENSMUSG00000069041.8
Ddx4	1.04	2.06	0.03	0.36	67.58	139.41	ENSMUSG00000021758.13
Arhgap20	1.04	2.06	0.01	0.23	4.18	8.61	ENSMUSG00000053199.13
Amer2	1.04	2.06	0.04	0.43	13.81	28.47	ENSMUSG00000021986.7
Syngr4	1.04	2.06	0.04	0.44	118.87	245.06	ENSMUSG00000040231.15
Nanp, Ninl	1.04	2.06	0.02	0.33	6.97	14.38	ENSMUSG00000068115.13
Phlpp1	1.04	2.06	0.05	0.45	21.83	44.97	ENSMUSG00000044340.7
Smim24	1.04	2.06	0.04	0.43	459.10	945.48	ENSMUSG00000078439.9
Zfp41	1.04	2.06	0.03	0.39	6.84	14.08	ENSMUSG00000047003.14
Cdkn3	1.04	2.06	0.03	0.34	75.37	155.14	ENSMUSG00000037628.9
Igbp1b	1.04	2.06	0.04	0.43	13.18	27.12	ENSMUSG00000046717.5
Rora	1.04	2.06	0.05	0.46	6.20	12.75	ENSMUSG00000032238.17
Pabpc2	1.04	2.06	0.03	0.38	136.13	280.08	ENSMUSG00000051732.2
Asap1	1.04	2.06	0.02	0.32	24.30	49.98	ENSMUSG00000022377.16
Grid1	1.64	2.06	0.02	0.29	1.00	3.10	ENSMUSG00000041078.6
Armc4	1.04	2.06	0.04	0.40	12.04	24.76	ENSMUSG00000061802.5
Rnf19a	1.04	2.06	0.04	0.41	57.75	118.71	ENSMUSG00000098457.1
Bbs7	1.04	2.06	0.02	0.31	14.85	30.52	ENSMUSG00000037325.10
Lancl1	1.04	2.05	0.03	0.35	108.76	223.47	ENSMUSG00000026000.16
Ccdc7a, Gm3952	1.04	2.05	0.04	0.40	10.99	22.57	ENSMUSG00000025808.16
Ube2t	1.04	2.05	0.05	0.44	23.87	49.03	ENSMUSG00000026429.8
Sycp1	1.04	2.05	0.03	0.36	24.34	49.97	ENSMUSG00000027855.13
Catsper4, Cnksr1	1.04	2.05	0.03	0.36	37.12	76.20	ENSMUSG00000028841.14
Socs7	1.04	2.05	0.03	0.35	22.01	45.18	ENSMUSG00000038485.5
Gzmn	1.04	2.05	0.04	0.44	16.95	34.77	ENSMUSG00000015443.8
Abca16	1.04	2.05	0.03	0.35	8.58	17.60	ENSMUSG00000051900.12
Ssx2ip	1.04	2.05	0.02	0.32	63.57	130.41	ENSMUSG00000036825.12
Spesp1	1.04	2.05	0.03	0.36	40.60	83.30	ENSMUSG00000046846.3
Plin2	1.03	2.05	0.05	0.45	21.14	43.32	ENSMUSG00000028494.12
Amhr2	1.03	2.05	0.03	0.33	29.40	60.23	ENSMUSG00000023047.10
Arhgap19	1.03	2.05	0.03	0.34	11.24	23.00	ENSMUSG00000025154.14
Cox8c	1.03	2.04	0.04	0.41	502.92	1028.14	ENSMUSG00000043319.4
4931429I11Rik	1.03	2.04	0.04	0.42	13.53	27.66	ENSMUSG00000032023.7
1700080E11Rik	1.03	2.04	0.02	0.33	75.00	153.32	ENSMUSG00000032566.4
Comp	1.03	2.04	0.03	0.35	47.51	97.12	ENSMUSG00000031849.8
Cenpf	1.03	2.04	0.02	0.32	7.50	15.33	ENSMUSG00000026605.14
Txnrd3	1.03	2.04	0.03	0.37	81.84	167.17	ENSMUSG00000000811.13
Umod	1.03	2.04	0.05	0.46	2.23	4.56	ENSMUSG00000030963.6

Slain2	1.03	2.04	0.03	0.34	44.10	90.02	ENSMUSG00000036087.18
Ttyh2	1.03	2.04	0.02	0.31	3.41	6.96	ENSMUSG00000034714.9
Serf1	1.03	2.04	0.03	0.35	341.74	697.15	ENSMUSG00000021643.14
Rangap1	1.03	2.04	0.03	0.34	59.08	120.43	ENSMUSG00000022391.15
Ttc9	1.03	2.04	0.04	0.41	9.32	19.00	ENSMUSG00000042734.5
4930415O20Rik	1.03	2.04	0.05	0.47	47.38	96.51	ENSMUSG00000022993.6
Cep55	1.03	2.04	0.05	0.46	5.14	10.46	ENSMUSG00000024989.14
Sel1l2	1.03	2.04	0.04	0.39	14.31	29.11	ENSMUSG00000074764.11
Serpine1	1.87	2.03	0.01	0.18	0.64	2.33	ENSMUSG00000037411.10
Birc5	1.02	2.03	0.04	0.40	25.02	50.88	ENSMUSG00000017716.15
BC022687	1.02	2.03	0.03	0.34	25.12	51.08	ENSMUSG00000037594.10
Ankrd7	1.02	2.03	0.04	0.41	44.42	90.12	ENSMUSG00000029517.13
Slc6a16	1.02	2.03	0.03	0.36	9.65	19.57	ENSMUSG00000094152.3
Gk2	1.02	2.03	0.04	0.41	140.59	285.10	ENSMUSG00000050553.3
Zp3r	1.02	2.03	0.04	0.41	25.75	52.23	ENSMUSG00000042554.10
Kcng1	1.02	2.03	0.05	0.46	2.76	5.59	ENSMUSG00000074575.4
Ppm1d	1.02	2.03	0.03	0.36	64.95	131.63	ENSMUSG00000020525.17
1700017B05Rik	1.02	2.03	0.02	0.29	16.52	33.47	ENSMUSG00000032300.6
Ccdc113	1.02	2.03	0.02	0.33	75.22	152.37	ENSMUSG00000036598.3
Wdr63	1.02	2.03	0.04	0.41	20.16	40.83	ENSMUSG00000043020.13
Ttc25	1.02	2.03	0.04	0.44	49.94	101.14	ENSMUSG00000006784.14
Cpt1c	1.02	2.02	0.03	0.38	14.52	29.40	ENSMUSG00000007783.9
Gm595	1.02	2.02	0.04	0.44	8.31	16.81	ENSMUSG00000079606.1
Lrrc23	1.02	2.02	0.03	0.38	49.15	99.41	ENSMUSG00000030125.11
Cfap74	1.02	2.02	0.04	0.41	13.81	27.93	ENSMUSG00000078490.9
Ttc21b	1.02	2.02	0.02	0.33	17.69	35.76	ENSMUSG00000034848.17
Fam183b	1.02	2.02	0.03	0.34	81.88	165.49	ENSMUSG00000049154.11
Susd3	1.02	2.02	0.03	0.36	12.12	24.50	ENSMUSG00000021384.14
Tekt3	1.01	2.02	0.03	0.35	56.48	114.13	ENSMUSG00000042189.5
Pbp2	1.01	2.02	0.04	0.42	73.86	149.20	ENSMUSG00000047104.5
Dcaf5	1.01	2.02	0.02	0.33	20.81	42.02	ENSMUSG00000049106.6
Arhgap29	1.01	2.02	0.03	0.36	44.58	90.00	ENSMUSG00000039831.16
Ttll9	1.01	2.02	0.03	0.37	49.81	100.51	ENSMUSG00000074673.15
Tmcc1	1.01	2.02	0.03	0.36	29.45	59.41	ENSMUSG00000030126.17
Rps6ka1	1.01	2.02	0.03	0.37	11.87	23.94	ENSMUSG00000003644.17
Ddx20	1.01	2.02	0.04	0.42	48.20	97.20	ENSMUSG00000027905.15
Taf7l	1.01	2.02	0.03	0.36	6.86	13.83	ENSMUSG00000009596.9
Fam53c	1.01	2.02	0.04	0.40	29.38	59.22	ENSMUSG00000034300.16
Urb1	1.01	2.01	0.04	0.39	7.88	15.88	ENSMUSG00000039929.14
Espl1	1.01	2.01	0.04	0.43	7.69	15.48	ENSMUSG00000058290.3
Plac8l1	1.01	2.01	0.05	0.44	32.20	64.87	ENSMUSG00000059455.2
Efhb	1.01	2.01	0.04	0.40	16.15	32.52	ENSMUSG00000023931.4
Racgap1	1.01	2.01	0.01	0.25	11.55	23.26	ENSMUSG00000023015.13
Lats2, Xpo4	1.01	2.01	0.02	0.30	27.76	55.86	ENSMUSG00000021952.15
Git1	1.01	2.01	0.05	0.44	115.58	232.52	ENSMUSG00000011877.13
Cks2	1.01	2.01	0.03	0.34	51.43	103.45	ENSMUSG00000062248.5
Adam26a, Adam26b	1.01	2.01	0.03	0.39	13.48	27.12	ENSMUSG00000048516.2

Dnajc27	1.01	2.01	0.03	0.36	19.11	38.42	ENSMUSG00000020657.15
Sh3gl3	1.01	2.01	0.04	0.43	36.62	73.63	ENSMUSG00000030638.13
Stambpl1	1.01	2.01	0.05	0.46	16.76	33.69	ENSMUSG00000024776.17
Ccdc105	1.01	2.01	0.04	0.41	39.63	79.63	ENSMUSG00000078442.2
Zfp105	1.01	2.01	0.03	0.34	14.60	29.34	ENSMUSG00000057895.11
Ptgr1	1.01	2.01	0.04	0.41	3.92	7.88	ENSMUSG00000028378.6
Arsa	1.01	2.01	0.03	0.37	38.14	76.59	ENSMUSG00000022620.14
Ston1	1.01	2.01	0.03	0.33	8.78	17.62	ENSMUSG00000033855.15
Ttll6	1.01	2.01	0.04	0.43	25.81	51.80	ENSMUSG00000038756.13
Pknox2	1.00	2.01	0.05	0.44	14.90	29.90	ENSMUSG00000035934.15
Nol4l	1.00	2.01	0.05	0.44	35.65	71.53	ENSMUSG00000061411.12
1700011A15Rik	1.00	2.01	0.03	0.34	46.52	93.32	ENSMUSG00000063971.6
Pold3	1.00	2.01	0.02	0.27	36.68	73.56	ENSMUSG00000030726.16
Nlrp14	1.00	2.00	0.03	0.34	48.28	96.79	ENSMUSG00000016626.10
Zswim2	1.00	2.00	0.05	0.45	35.88	71.91	ENSMUSG00000034552.8
Hsf5	1.00	2.00	0.02	0.33	33.39	66.91	ENSMUSG00000070345.3
Fam76a	1.00	2.00	0.03	0.34	37.21	74.50	ENSMUSG00000028878.11
Adgb	1.00	2.00	0.05	0.46	10.30	20.61	ENSMUSG00000050994.20
Pbk	1.00	2.00	0.02	0.29	20.42	40.87	ENSMUSG00000022033.9
C1qtnf4	1.00	2.00	0.04	0.41	71.75	143.55	ENSMUSG00000040794.5
Ica1	1.00	2.00	0.03	0.37	46.46	92.94	ENSMUSG00000062995.12
Ccdc171	1.00	2.00	0.04	0.44	11.83	23.67	ENSMUSG00000052407.16
Lrriq1	1.00	2.00	0.03	0.39	6.59	13.18	ENSMUSG00000019892.12
Ipo13	1.00	2.00	0.04	0.41	32.73	65.43	ENSMUSG00000033365.14
Znhit2	1.00	2.00	0.03	0.35	130.12	260.06	ENSMUSG00000075227.6
Epb41l3	1.00	2.00	0.03	0.33	12.94	25.85	ENSMUSG00000024044.16
Tmem232	1.00	2.00	0.04	0.40	24.70	49.35	ENSMUSG00000045036.14
Rnf138	1.00	2.00	0.04	0.39	63.23	126.34	ENSMUSG00000024317.14
Hyal4,Hyal6	1.00	2.00	0.04	0.41	10.23	20.45	ENSMUSG00000029680.2
Tdrd9	1.00	2.00	0.03	0.38	17.51	34.97	ENSMUSG00000054003.13
Dnajb2	1.00	2.00	0.03	0.35	75.69	151.16	ENSMUSG00000026203.16
Spef2	1.00	2.00	0.05	0.46	6.48	12.94	ENSMUSG00000072663.12
Smap1	1.00	2.00	0.04	0.41	71.99	143.69	ENSMUSG00000026155.13
Fam71f2, Hilpda	1.00	2.00	0.04	0.43	181.97	363.18	ENSMUSG00000043421.8
Ckap2	1.00	2.00	0.05	0.46	3.29	6.57	ENSMUSG00000037725.7
Cfap97	1.00	2.00	0.05	0.44	57.55	114.84	ENSMUSG00000031631.15
Spice1	1.00	1.99	0.04	0.42	5.84	11.64	ENSMUSG00000043065.12
Sipa1l2	0.99	1.99	0.04	0.39	4.81	9.59	ENSMUSG00000001995.8
1700040L02Rik	0.99	1.99	0.03	0.37	48.55	96.70	ENSMUSG00000019945.9
Trpd52l3	0.99	1.99	0.02	0.33	18.66	37.16	ENSMUSG00000024815.4
Oasl1	2.03	1.99	0.04	0.41	0.47	1.93	ENSMUSG00000041827.15
Amigo3, Cdhr4	0.99	1.99	0.03	0.37	180.07	358.34	ENSMUSG00000032596.14
Ttc30a1, Ttc30a2	0.99	1.99	0.04	0.42	28.49	56.68	ENSMUSG00000075271.4
Tdp1	0.99	1.99	0.04	0.42	26.59	52.89	ENSMUSG00000021177.15
Smg9	0.99	1.99	0.04	0.39	45.61	90.69	ENSMUSG00000002210.11
Id4	0.99	1.99	0.03	0.38	9.54	18.97	ENSMUSG00000021379.1
Dnajc9	0.99	1.99	0.02	0.29	25.94	51.55	ENSMUSG00000021811.6

Rab31	0.99	1.99	0.02	0.32	10.64	21.14	ENSMUSG00000056515.8
Triml1	0.99	1.99	0.04	0.41	16.90	33.58	ENSMUSG00000031651.4
Gpat4	0.99	1.99	0.03	0.38	88.84	176.52	ENSMUSG00000031545.5
Atp6ap1l	0.99	1.99	0.05	0.47	32.97	65.51	ENSMUSG00000078958.9
Ppp4r4	0.99	1.99	0.03	0.37	8.36	16.60	ENSMUSG00000021209.12
Sdad1	0.99	1.99	0.04	0.42	19.57	38.86	ENSMUSG00000029415.4
4930544G11Rik	0.99	1.98	0.03	0.37	60.79	120.59	ENSMUSG00000036463.8
Nme5	0.99	1.98	0.04	0.39	66.14	131.14	ENSMUSG00000035984.14
Hepacam	0.99	1.98	0.04	0.44	4.17	8.27	ENSMUSG00000046240.7
Kifap3	0.99	1.98	0.03	0.37	40.97	81.14	ENSMUSG00000026585.13
Msi1	0.98	1.98	0.03	0.37	11.35	22.46	ENSMUSG00000054256.11
Gfra4	0.98	1.98	0.04	0.44	9.17	18.14	ENSMUSG00000027316.15
Tex21	0.98	1.98	0.04	0.43	34.12	67.50	ENSMUSG00000021056.7
Ccdc178	0.98	1.98	0.04	0.43	15.20	30.06	ENSMUSG00000024306.12
Specc1	0.98	1.98	0.04	0.43	5.47	10.83	ENSMUSG00000042331.13
Ftmt	0.98	1.98	0.04	0.42	25.94	51.31	ENSMUSG00000024510.5
Nup205	0.98	1.98	0.02	0.26	32.87	65.00	ENSMUSG00000038759.15
Eif1b	0.98	1.98	0.03	0.35	73.77	145.85	ENSMUSG00000006941.4
Btbd10	0.98	1.98	0.03	0.38	28.27	55.86	ENSMUSG00000038187.14
Cfap161	0.98	1.97	0.03	0.36	38.32	75.66	ENSMUSG00000011154.17
Aldh1a2	0.98	1.97	0.02	0.31	21.95	43.34	ENSMUSG00000013584.5
Ssh2	0.98	1.97	0.03	0.36	19.71	38.89	ENSMUSG00000037926.15
Mapk6	0.98	1.97	0.04	0.39	74.66	147.28	ENSMUSG00000042688.16
Arid3b	0.98	1.97	0.03	0.39	9.20	18.14	ENSMUSG00000004661.15
Lrrc34	0.98	1.97	0.05	0.46	64.63	127.36	ENSMUSG00000027702.7
Flcn, Pld6	0.98	1.97	0.03	0.37	23.09	45.50	ENSMUSG00000032633.12
Ak7	0.98	1.97	0.03	0.34	21.07	41.51	ENSMUSG00000041323.5
Zfp35	0.98	1.97	0.02	0.31	22.94	45.18	ENSMUSG00000063281.9
Toporsl	0.98	1.97	0.03	0.36	29.93	58.88	ENSMUSG00000028314.6
Ap2b1	0.98	1.97	0.04	0.42	100.45	197.57	ENSMUSG00000035152.14
Ccdc42	0.97	1.96	0.05	0.45	39.95	78.45	ENSMUSG00000045915.15
Ap1m1	0.97	1.96	0.02	0.32	77.55	152.23	ENSMUSG00000003033.14
Fam20a	0.97	1.96	0.04	0.44	10.30	20.16	ENSMUSG00000020614.13
Gm42421	0.97	1.96	0.04	0.42	20.30	39.75	ENSMUSG00000075569.9
Gas7	0.97	1.96	0.04	0.42	7.61	14.89	ENSMUSG00000033066.15
Ttc12	0.97	1.96	0.03	0.38	10.43	20.42	ENSMUSG00000040219.4
Usp1	0.97	1.96	0.05	0.45	73.33	143.46	ENSMUSG00000028560.11
Arl2bp	0.97	1.96	0.03	0.38	78.60	153.70	ENSMUSG00000031776.16
Mapk8ip2	0.97	1.95	0.04	0.40	8.97	17.52	ENSMUSG00000022619.5
Per1	0.97	1.95	0.03	0.33	19.55	38.18	ENSMUSG00000020893.17
Sqrdl	0.96	1.95	0.03	0.37	124.29	242.57	ENSMUSG00000005803.14
Usp15	0.96	1.95	0.04	0.40	47.04	91.80	ENSMUSG00000020124.9
Cdkn2aip	0.96	1.95	0.03	0.37	19.08	37.22	ENSMUSG00000038069.5
Vps13a	0.96	1.95	0.05	0.45	11.96	23.32	ENSMUSG00000046230.9
Man2b2	0.96	1.95	0.03	0.36	25.46	49.65	ENSMUSG00000029119.9
Atf1	0.96	1.95	0.05	0.47	46.84	91.27	ENSMUSG00000023027.12
Abcf2	0.96	1.95	0.04	0.44	54.15	105.40	ENSMUSG00000028953.10

Shcbp1	0.96	1.95	0.04	0.41	12.15	23.65	ENSMUSG00000022322.8
Dyrk3	0.96	1.95	0.03	0.38	36.74	71.48	ENSMUSG00000016526.8
Fbxo34	0.96	1.94	0.04	0.40	11.57	22.49	ENSMUSG00000037536.13
Mllt10	0.96	1.94	0.05	0.45	31.43	61.10	ENSMUSG00000026743.16
Gdpd1	0.96	1.94	0.03	0.39	22.82	44.36	ENSMUSG00000061666.6
Crip3, Zfp318	0.96	1.94	0.03	0.33	27.32	53.00	ENSMUSG00000023968.15
Zfp956	0.95	1.94	0.02	0.32	25.82	49.99	ENSMUSG00000072653.12
Cenpe	0.95	1.94	0.04	0.39	8.26	15.99	ENSMUSG00000045328.11
Dpy19l1	0.95	1.93	0.04	0.41	9.65	18.65	ENSMUSG00000043067.15
Gm3424, Gm8237	1.62	1.93	0.03	0.39	0.82	2.52	ENSMUSG00000090512.8
Cfap126	0.95	1.93	0.04	0.39	82.30	159.02	ENSMUSG00000026649.14
Htatip2	0.95	1.93	0.05	0.44	14.13	27.22	ENSMUSG00000039745.8
Azin2	0.94	1.92	0.04	0.39	52.71	101.47	ENSMUSG00000028789.16
Sfmbt2	0.94	1.92	0.05	0.47	6.22	11.97	ENSMUSG00000061186.15
Rasl2-9	0.94	1.92	0.04	0.43	55.26	106.28	ENSMUSG00000083649.5
Bbs5	0.94	1.92	0.03	0.38	29.61	56.92	ENSMUSG00000063145.10
Sccpdh	0.94	1.92	0.04	0.42	107.15	205.90	ENSMUSG00000038936.13
Lrrc28	0.94	1.92	0.04	0.44	56.49	108.43	ENSMUSG00000030556.13
Lrguk	0.94	1.92	0.05	0.46	7.27	13.94	ENSMUSG00000056215.12
Map10	0.94	1.91	0.03	0.35	7.85	15.01	ENSMUSG00000050930.5
Ints10	0.94	1.91	0.04	0.42	38.78	74.20	ENSMUSG00000031864.15
Cerk	0.94	1.91	0.04	0.43	14.13	27.02	ENSMUSG00000035891.16
Cntln	0.94	1.91	0.03	0.35	5.62	10.74	ENSMUSG00000038070.15
Dnajc15	0.93	1.91	0.03	0.35	151.80	290.21	ENSMUSG00000022013.3
Drc1	0.93	1.91	0.04	0.39	26.95	51.48	ENSMUSG00000073102.7
Rtp4	2.26	1.91	0.01	0.20	0.32	1.51	ENSMUSG00000033355.5
Slfn2	1.67	1.91	0.03	0.38	0.72	2.28	ENSMUSG00000072620.3
Fignl1	0.93	1.91	0.04	0.43	8.15	15.56	ENSMUSG00000035455.12
Coprs	0.93	1.91	0.05	0.45	29.86	56.91	ENSMUSG00000031458.7
Casc5	0.93	1.90	0.05	0.46	2.83	5.40	ENSMUSG00000027326.13
Mdc1	0.93	1.90	0.04	0.43	22.17	42.16	ENSMUSG00000061607.14
Mrgbp	0.93	1.90	0.04	0.40	25.66	48.78	ENSMUSG00000027569.15
Chchd6	0.93	1.90	0.04	0.43	79.28	150.69	ENSMUSG00000030086.16
Chd1l	0.93	1.90	0.03	0.35	19.65	37.35	ENSMUSG00000028089.5
Svbp	0.92	1.90	0.05	0.46	59.88	113.52	ENSMUSG00000028643.10
Dnaic2	0.92	1.90	0.04	0.41	15.34	29.07	ENSMUSG00000034706.16
Smim13	0.92	1.89	0.04	0.43	8.44	16.00	ENSMUSG00000091264.2
Rdh11	0.92	1.89	0.04	0.43	47.80	90.39	ENSMUSG00000066441.14
Bub1	0.92	1.89	0.05	0.45	11.20	21.18	ENSMUSG00000027379.13
Nupr1l	0.92	1.89	0.04	0.43	147.40	278.58	ENSMUSG00000095789.6
Lin7a	0.92	1.89	0.05	0.46	9.30	17.57	ENSMUSG00000019906.14
Ints6	0.92	1.89	0.05	0.45	17.80	33.60	ENSMUSG00000035161.6
Ahctf1, Gm1305	0.92	1.89	0.04	0.41	30.81	58.12	ENSMUSG00000026491.13
Gatm	0.92	1.89	0.04	0.40	20.31	38.32	ENSMUSG00000027199.14
Usp18	2.34	1.89	0.01	0.14	0.28	1.41	ENSMUSG00000030107.10
Top2a	0.91	1.88	0.04	0.39	19.50	36.74	ENSMUSG00000020914.17
Kat7	0.91	1.88	0.04	0.43	36.23	68.27	ENSMUSG00000038909.16

Zscan20	0.91	1.88	0.05	0.45	3.37	6.35	ENSMUSG00000061894.15
Bcap29	0.91	1.88	0.04	0.41	45.58	85.80	ENSMUSG00000020650.14
Ltn1	0.91	1.88	0.03	0.35	12.02	22.62	ENSMUSG00000052299.8
Elmo1	0.91	1.88	0.04	0.42	6.67	12.55	ENSMUSG00000041112.15
Ccnb2	0.91	1.88	0.05	0.44	45.95	86.43	ENSMUSG00000032218.6
Itgam	1.51	1.88	0.01	0.23	0.91	2.60	ENSMUSG00000030786.18
Wdr35	0.91	1.88	0.04	0.43	22.40	42.12	ENSMUSG00000066643.12
Umps	0.91	1.88	0.05	0.46	3.98	7.48	ENSMUSG00000022814.6
Rapgef5	0.91	1.88	0.04	0.42	4.26	8.00	ENSMUSG00000041992.8
Mtmr6	0.90	1.87	0.04	0.41	37.34	69.91	ENSMUSG00000021987.8
Insl6	0.90	1.87	0.03	0.39	83.75	156.71	ENSMUSG00000050957.4
Fam135a	0.90	1.87	0.04	0.44	16.71	31.25	ENSMUSG00000026153.15
Mzt2	0.90	1.87	0.04	0.44	57.87	108.19	ENSMUSG00000022671.12
Uchl3	0.90	1.86	0.04	0.40	51.74	96.45	ENSMUSG00000022111.8
Uri1	0.90	1.86	0.04	0.43	35.43	65.99	ENSMUSG00000030421.9
Bub1b	0.90	1.86	0.04	0.42	8.76	16.30	ENSMUSG00000040084.9
Itgb2	1.47	1.86	0.03	0.36	0.94	2.61	ENSMUSG00000000290.13
Tpx2	0.89	1.86	0.04	0.43	8.26	15.36	ENSMUSG00000027469.16
Glipr1l1	0.89	1.86	0.05	0.47	41.46	76.97	ENSMUSG00000020213.6
Bag6	0.89	1.86	0.04	0.42	186.76	346.67	ENSMUSG00000024392.17
Usp25	0.89	1.85	0.05	0.44	21.82	40.47	ENSMUSG00000022867.9
Sucla2	0.89	1.85	0.04	0.44	113.81	210.93	ENSMUSG00000022110.13
Cfap58	0.89	1.85	0.05	0.46	28.04	51.91	ENSMUSG00000046585.8
Sfmbt1	0.89	1.85	0.05	0.45	17.64	32.65	ENSMUSG00000006527.14
Cldn11	0.88	1.85	0.03	0.34	20.30	37.47	ENSMUSG00000037625.7
Zswim4	0.88	1.84	0.04	0.43	4.17	7.69	ENSMUSG00000035671.5
Nxt1	0.88	1.84	0.05	0.46	54.25	100.04	ENSMUSG00000036992.10
Ak1	0.88	1.84	0.04	0.40	59.69	110.02	ENSMUSG00000026817.14
Pacs1	0.88	1.84	0.04	0.44	30.00	55.16	ENSMUSG00000024855.9
Klhl25	0.88	1.84	0.04	0.39	12.22	22.46	ENSMUSG00000055652.14
Ctnnbl1	0.88	1.84	0.05	0.46	30.01	55.11	ENSMUSG00000027649.15
Kiz	0.88	1.84	0.03	0.39	60.10	110.35	ENSMUSG00000074749.10
Zfp608	0.88	1.83	0.05	0.44	8.86	16.26	ENSMUSG00000052713.8
Fam220a	0.87	1.83	0.04	0.42	38.87	71.26	ENSMUSG00000083012.8
Ttll4	0.87	1.83	0.05	0.46	22.54	41.28	ENSMUSG00000033257.14
Ank2	0.87	1.83	0.04	0.40	13.12	24.01	ENSMUSG00000032826.16
Optn	0.87	1.83	0.05	0.46	33.91	62.06	ENSMUSG00000026672.11
Myef2	0.87	1.83	0.05	0.46	10.95	20.02	ENSMUSG00000027201.16
Ell2	0.87	1.82	0.05	0.44	25.52	46.54	ENSMUSG00000001542.6
Map4	0.86	1.82	0.04	0.41	40.84	74.20	ENSMUSG00000032479.15
Uchl1	0.86	1.82	0.04	0.43	17.66	32.05	ENSMUSG00000029223.12
Myo7a	0.86	1.81	0.04	0.42	15.04	27.29	ENSMUSG00000030761.15
Tln2	0.86	1.81	0.05	0.47	15.51	28.08	ENSMUSG00000052698.14
4930539E08Rik	1.42	1.81	0.03	0.34	0.93	2.50	ENSMUSG00000048905.4
Phf13	0.86	1.81	0.05	0.46	18.12	32.79	ENSMUSG00000047777.9
Dgat2	0.85	1.80	0.03	0.39	52.23	94.26	ENSMUSG00000030747.5
Itga11	1.39	1.80	0.03	0.34	0.98	2.58	ENSMUSG00000032243.8

Prdx4	0.85	1.80	0.04	0.42	60.41	108.91	ENSMUSG00000025289.15
Wwp2	0.85	1.80	0.04	0.41	19.63	35.33	ENSMUSG00000031930.10
Lgals8	0.85	1.80	0.03	0.36	57.81	104.04	ENSMUSG00000057554.13
4930402H24Rik	0.85	1.80	0.02	0.31	13.15	23.65	ENSMUSG00000085566.1
Ptcd3	0.84	1.79	0.04	0.41	16.22	29.07	ENSMUSG00000063884.6
Mfsd2b	1.70	1.79	0.02	0.28	0.55	1.77	ENSMUSG00000037336.14
Thoc5	0.84	1.79	0.05	0.46	28.52	51.02	ENSMUSG00000034274.11
Ccr5	1.88	1.79	0.00	0.08	0.42	1.53	ENSMUSG00000079227.9
Clasp1	0.84	1.79	0.05	0.46	16.88	30.14	ENSMUSG00000064302.13
Vps26a	0.84	1.78	0.04	0.42	55.14	98.42	ENSMUSG00000020078.15
Dr1	0.83	1.78	0.03	0.36	20.30	36.20	ENSMUSG00000029265.4
Btaf1	0.83	1.78	0.04	0.44	13.83	24.65	ENSMUSG00000040565.7
Vim	0.83	1.78	0.04	0.40	39.57	70.48	ENSMUSG00000026728.9
Rnf216	0.83	1.78	0.04	0.43	23.34	41.58	ENSMUSG00000045078.12
Tmem120a	0.83	1.78	0.04	0.44	33.44	59.52	ENSMUSG00000039886.8
Cxcl1	2.96	1.78	0.01	0.24	0.13	1.01	ENSMUSG00000029380.11
Ccdc109b	1.36	1.77	0.05	0.45	0.97	2.49	ENSMUSG00000027994.14
Ccl22	1.64	1.77	0.02	0.28	0.58	1.80	ENSMUSG00000031779.3
Sepw1	0.82	1.76	0.04	0.42	113.66	200.34	ENSMUSG00000041571.9
Usp32	0.82	1.76	0.05	0.46	23.76	41.86	ENSMUSG00000000804.14
Pnma2	1.42	1.75	0.02	0.29	0.81	2.16	ENSMUSG00000046204.13
BC027072	1.37	1.74	0.03	0.34	0.88	2.28	ENSMUSG00000044375.7
Oit3	1.75	1.74	0.02	0.28	0.46	1.54	ENSMUSG00000009654.4
Myoz2	1.89	1.73	0.05	0.45	0.37	1.36	ENSMUSG00000028116.13
Gbp3	1.65	1.72	0.01	0.17	0.51	1.59	ENSMUSG00000028268.14
Stk32a	2.20	1.69	0.02	0.33	0.24	1.08	ENSMUSG00000039954.8
Gpr37	1.32	1.68	0.03	0.37	0.85	2.11	ENSMUSG00000039904.9
Slc5a2	1.75	1.67	0.01	0.21	0.39	1.33	ENSMUSG00000030781.13
Pirb	1.98	1.67	0.03	0.35	0.29	1.16	ENSMUSG00000058818.13
Plek	1.45	1.66	0.02	0.33	0.61	1.67	ENSMUSG00000020120.15
Slc6a14	1.62	1.63	0.04	0.41	0.43	1.33	ENSMUSG00000031089.7
Elavl2	1.31	1.62	0.03	0.36	0.72	1.78	ENSMUSG00000008489.18
Sall4	1.81	1.62	0.01	0.22	0.33	1.15	ENSMUSG00000027547.17
Mx1	1.72	1.61	0.04	0.41	0.36	1.20	ENSMUSG00000000386.14
Ugt8a	1.44	1.61	0.04	0.43	0.55	1.49	ENSMUSG00000032854.12
Fam64a	1.67	1.60	0.02	0.26	0.38	1.20	ENSMUSG00000020808.3
Adgrg7	1.92	1.59	0.02	0.33	0.27	1.01	ENSMUSG00000022755.4
Myo1f	1.72	1.57	0.03	0.35	0.33	1.08	ENSMUSG00000024300.16
Thbs1	1.12	1.57	0.03	0.39	0.94	2.05	ENSMUSG00000040152.8
Sspo	1.57	1.57	0.03	0.37	0.40	1.20	ENSMUSG00000029797.12
Hrh4	1.47	1.56	0.04	0.42	0.46	1.28	ENSMUSG00000037346.4
Gm3127, Gm8108	1.50	1.53	0.05	0.46	0.41	1.16	ENSMUSG00000090764.8
Syt14	1.63	1.52	0.01	0.20	0.33	1.01	ENSMUSG00000016200.13

a: The uncorrected p-value of the test statistic.

b: The FDR-adjusted p-value of the test statistic.
