# Supplementary figures and images for: S100A4+ macrophages facilitate zika virus invasion and persistence in the seminiferous tubules via interferon-gamma mediation
Source: PLoS Pathog. 2020 Dec 14;16(12):e1009019. doi: 10.1371/journal.ppat.1009019 (PMC7769614; doi:10.1371/journal.ppat.1009019)

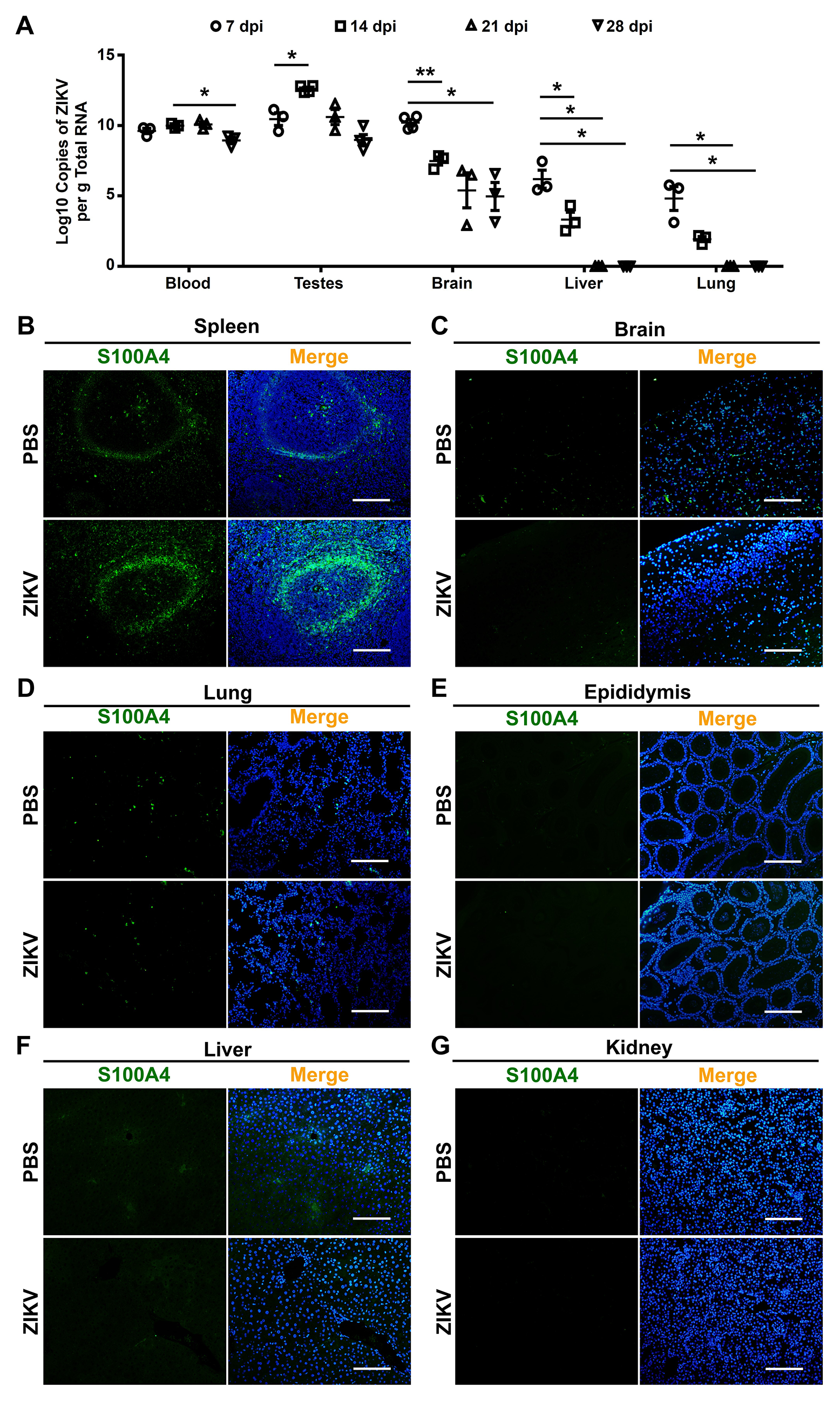

Supplement: S1 Fig — (A) ZIKV RNA in the whole blood, testes, brain, liver and lung obtained from ZIKV-infected A6 mice from 7 to 28 dpi was measured using RT-qPCR and shown as means ± SEM (n = 3–4 mice for each group), and analyzed using the Student’s t test. *p < 0.05, **p < 0.01. (B-G) Male A6 mice were challenged with ZIKV or injected with PBS. Main organs including (B) spleen, (C) brain, (D) lung, (E) epididymis, (F) liver and (G) kidney were isolated at 14 dpi and analyzed using immunofluorescence staining with anti-S100A4 antibody. Nuclei were shown with DAPI. Scale bar, 25 μm. Related to Fig 1D. (TIF) [file ppat.1009019.s002.tif]

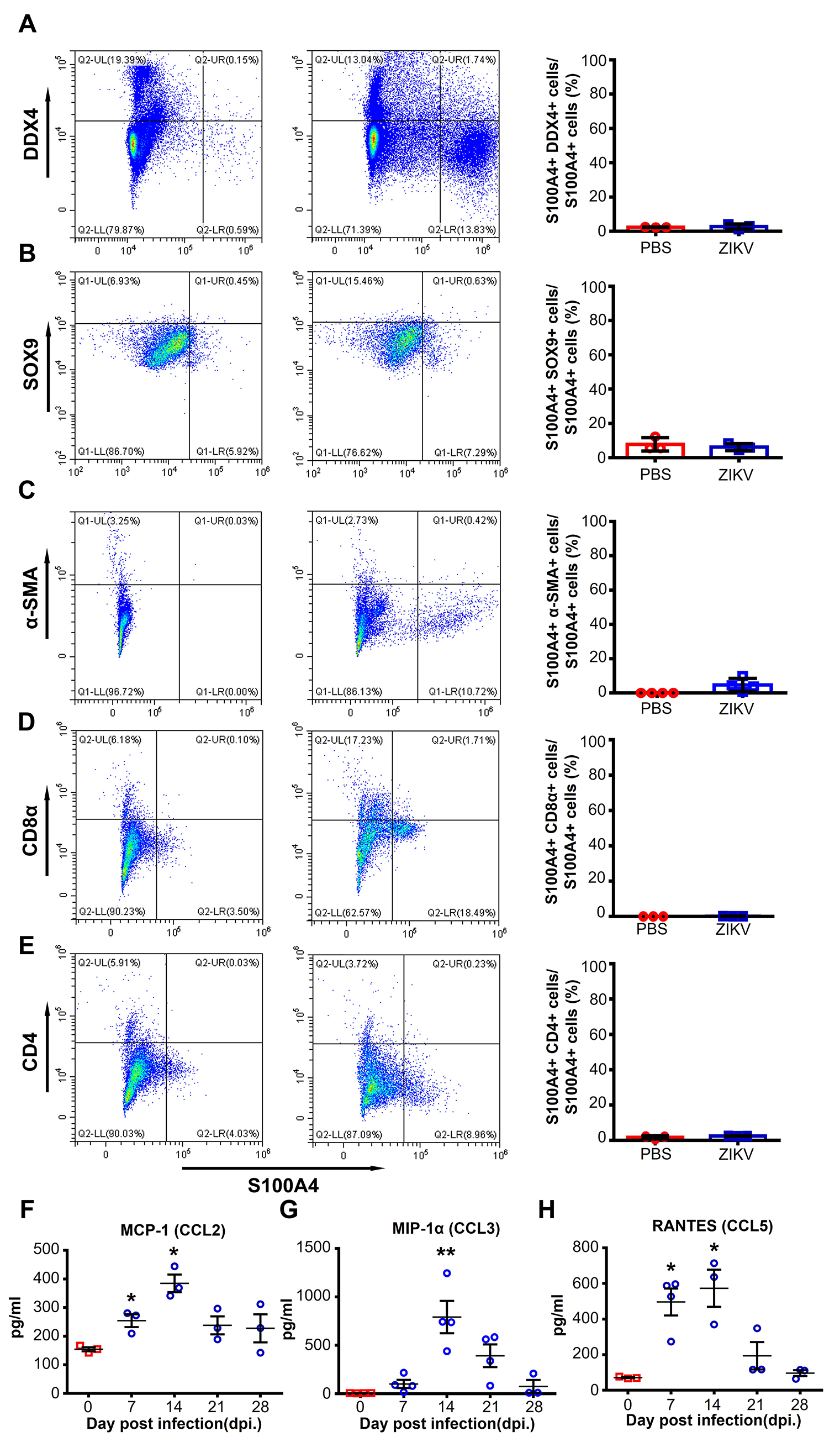

Supplement: S2 Fig — Testicular cells from ZIKV-infected (14 dpi) or PBS-injected A6 mice were subjected to flow cytometry analysis with anti-S100A4 antibody and (A) anti-DDX4 antibody, (B) anti-SOX9 antibody, (C) anti-α-SMA antibody, (D) anti-CD8α antibody, or (E) anti-CD4 antibody. Percentage of S100A4+ cells in each population were shown as means ± SEM. (n = 3–4 mice for each group). Related to Fig 1E and 1F. (F-H) Expression of 3 macrophages-recruitment related chemokines in ZIKV-infected testes of A6 mice from 7 to 28 dpi was measured using Luminex assay and shown as means ± SEM. (n = 3–4 mice for each time point). Concentration of chemokines were analyzed using the Student’s t test. *p < 0.05 versus 0 dpi, **p < 0.01 versus 0 dpi. (TIF) [file ppat.1009019.s003.tif]

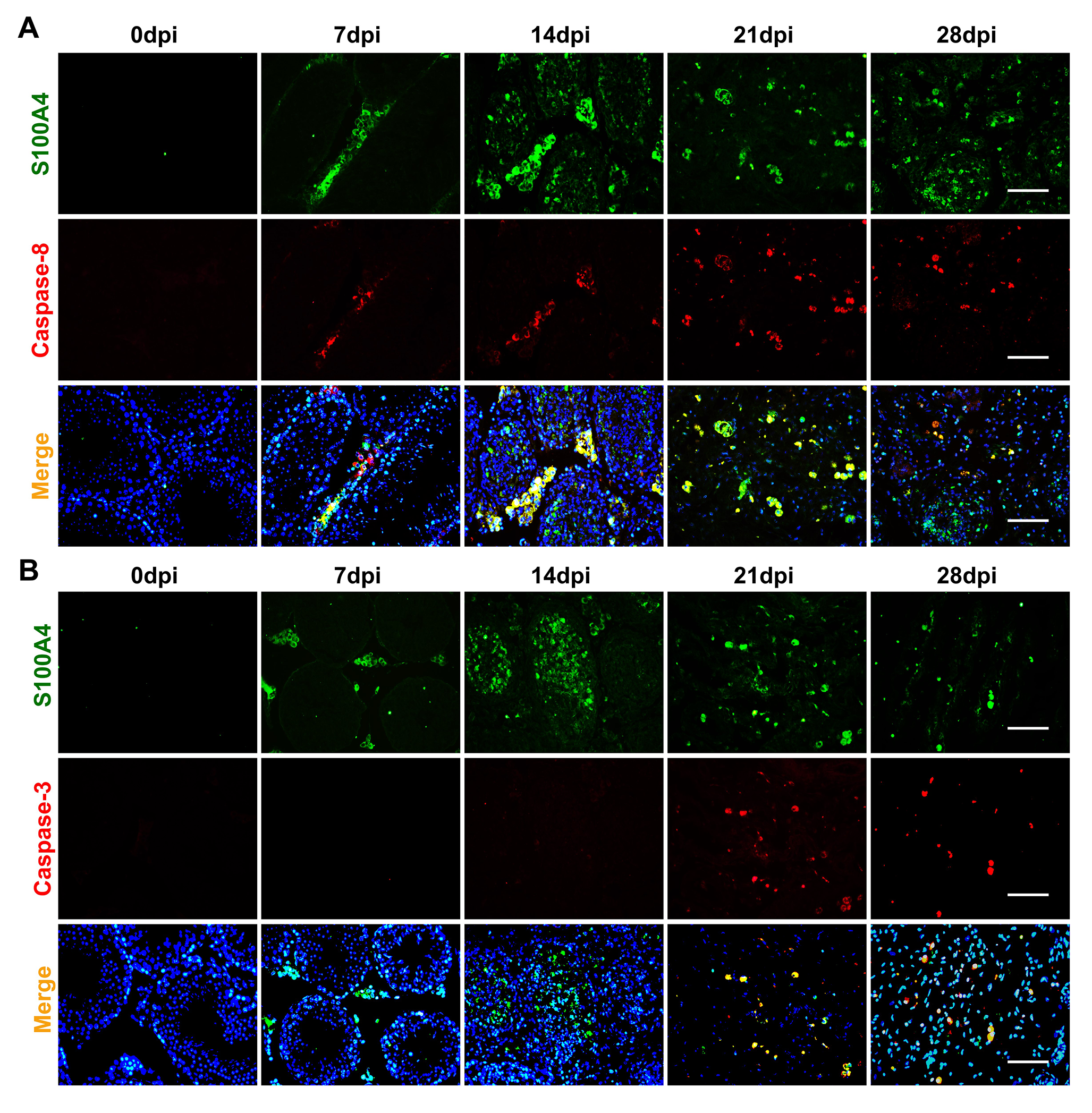

Supplement: S3 Fig — Testes from ZIKV-infected A6 mice were isolated at indicated time points and subjected to co-immunofluorescence staining with anti-S100A4 antibody and (A) anti-caspase-8 antibody, or (B) anti-caspase-3 antibody. Nuclei were shown with DAPI. Scale bar, 25 μm. The quantification of these results was shown in Fig 3D. (TIF) [file ppat.1009019.s004.tif]

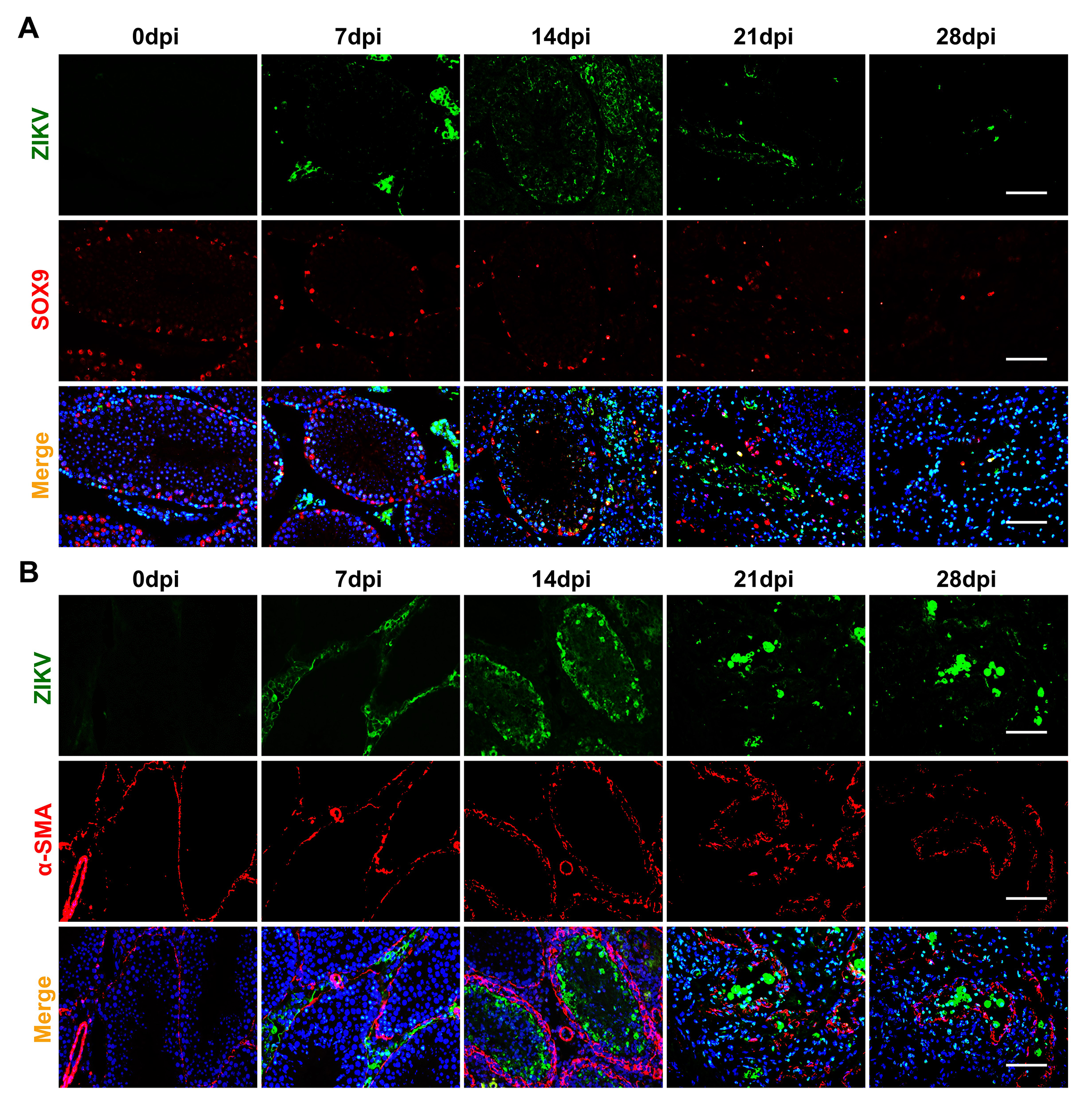

Supplement: S4 Fig — Testes from ZIKV-infected A6 mice were isolated at indicated time points and subjected to co-immunofluorescence staining with anti-ZIKV antibody and (A) anti-SOX9 antibody, or (B) anti-α-SMA antibody. Nuclei were shown with DAPI. Scale bar, 25 μm. The quantification of these results was shown in Fig 4C. (TIF) [file ppat.1009019.s005.tif]

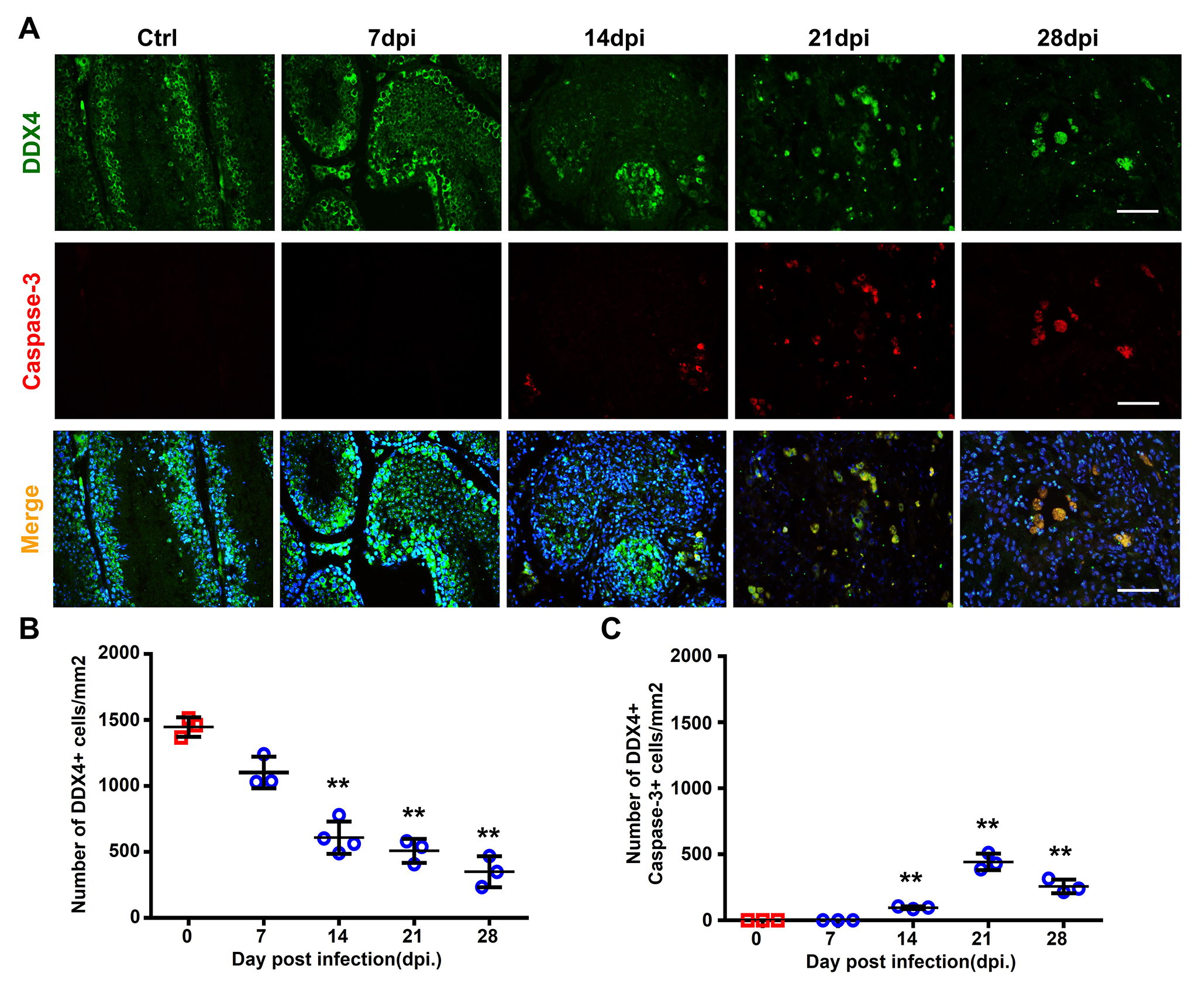

Supplement: S5 Fig — Testes from ZIKV-infected A6 mice were isolated at indicated time points and subjected to co-immunofluorescence staining with anti-DDX4 antibody and anti-caspase-3 antibody (A). Nuclei were shown with DAPI. Scale bar, 25 μm. Number of DDX4+ spermatogonium cells (B) and DDX4+ caspase-3+ cells (C) at 7–28 dpi was analyzed by Image J and shown as means ± SEM (n = 3 mice for each group). The number of indicated cells were analyzed using the Student’s t test. *p < 0.05 versus 0 dpi, **p < 0.01 versus 0 dpi. (TIF) [file ppat.1009019.s006.tif]

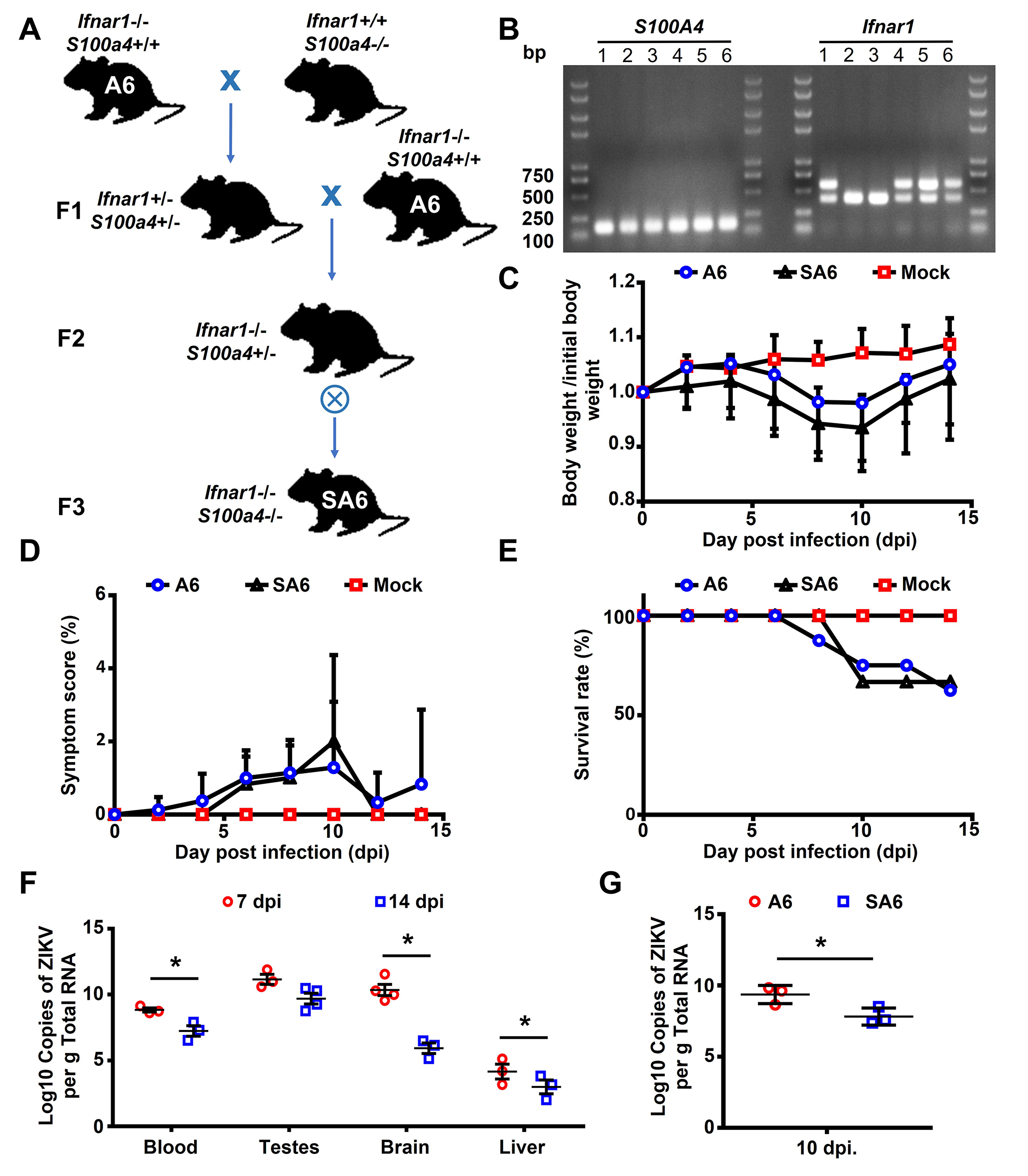

Supplement: S6 Fig — (A) SA6 mice were generated by mating S100A4 deficient mice with A6 mice. (B) Identification of SA6 mice with genomic PCR. (C-E) 6–8 weeks old SA6 male mice were i.p. challenged with 104 pfu of ZIKV. Mock mice were injected with PBS. Body weights (C), symptom scores (D) and survival rates (E) were monitored daily. (n = 6–8 mice for each group). (F) ZIKV RNA in whole blood and testes, brain, and liver from ZIKV-infected SA6 mice at 7 and 14 dpi were measured using RT-qPCR and shown as means ± SEM (n = 3–4 for each group). (G) ZIKV RNA in semen from ZIKV-infected A6 and SA6 mice at 10 dpi were determined by RT-qPCR. Results were shown as means ± SEM. (n = 3 mice for each group). Comparison in body weight and symptom score among three groups were analyzed using repeated-measures ANOVA. *p < 0.05, **p < 0.01. ZIKV RNA loads were analyzed using the Student’s t test. *p < 0.05, **p < 0.01. (TIF) [file ppat.1009019.s007.tif]

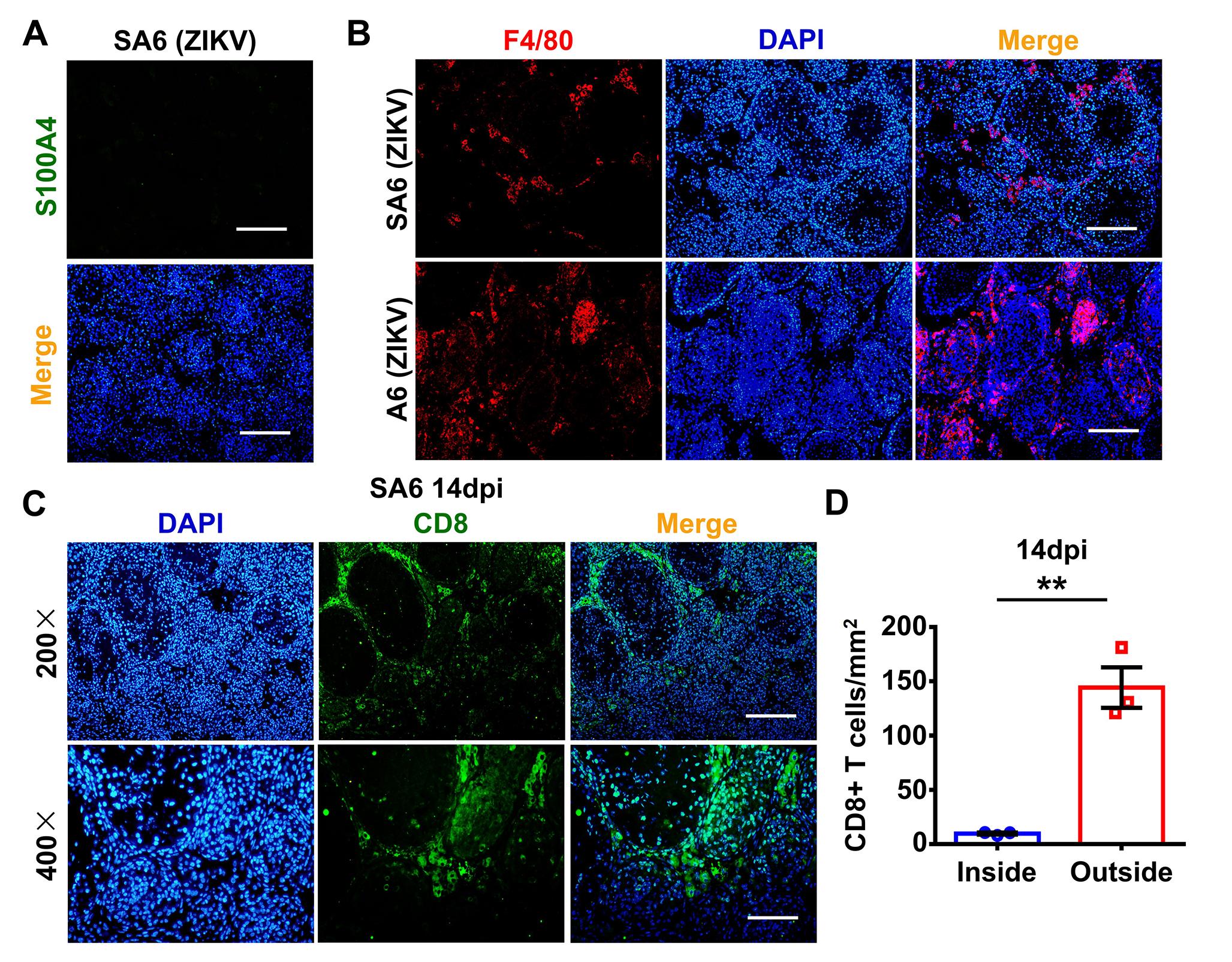

Supplement: S7 Fig — (A) Testicular sections from ZIKV-infected SA6 mice at 14 dpi were analyzed by immunofluorescence staining with anti-S100A4 antibody. Nuclei were shown with DAPI. Scale bar, 25 μm. (B) Distribution of F4/80+ macrophages in testes from ZIKV-infected A6 and SA6 mice. Testicular sections from ZIKV-infected SA6 and A6 mice at 14 dpi were analyzed by immunofluorescence staining with anti-F4/80 antibody. Nuclei were shown with DAPI. Scale bar, 25 μm. The quantification of these results was shown in Fig 5C. (C) Distribution of CD8+ T cells in testes from ZIKV-infected SA6 mice. Testes from ZIKV-infected SA6 mice at 14 dpi were isolated and subjected to immunofluorescence staining with anti-CD8α antibodies. The staining intensity of CD8+ cells inside and outside seminiferous tubules were quantified respectively and shown as means ± SEM (D). (n = 3 mice for each group). Nuclei are visualized with DAPI. Scale bar, 25 μm. Data were analyzed using the Student’s t test. *p < 0.05, **p < 0.01. (TIF) [file ppat.1009019.s008.tif]

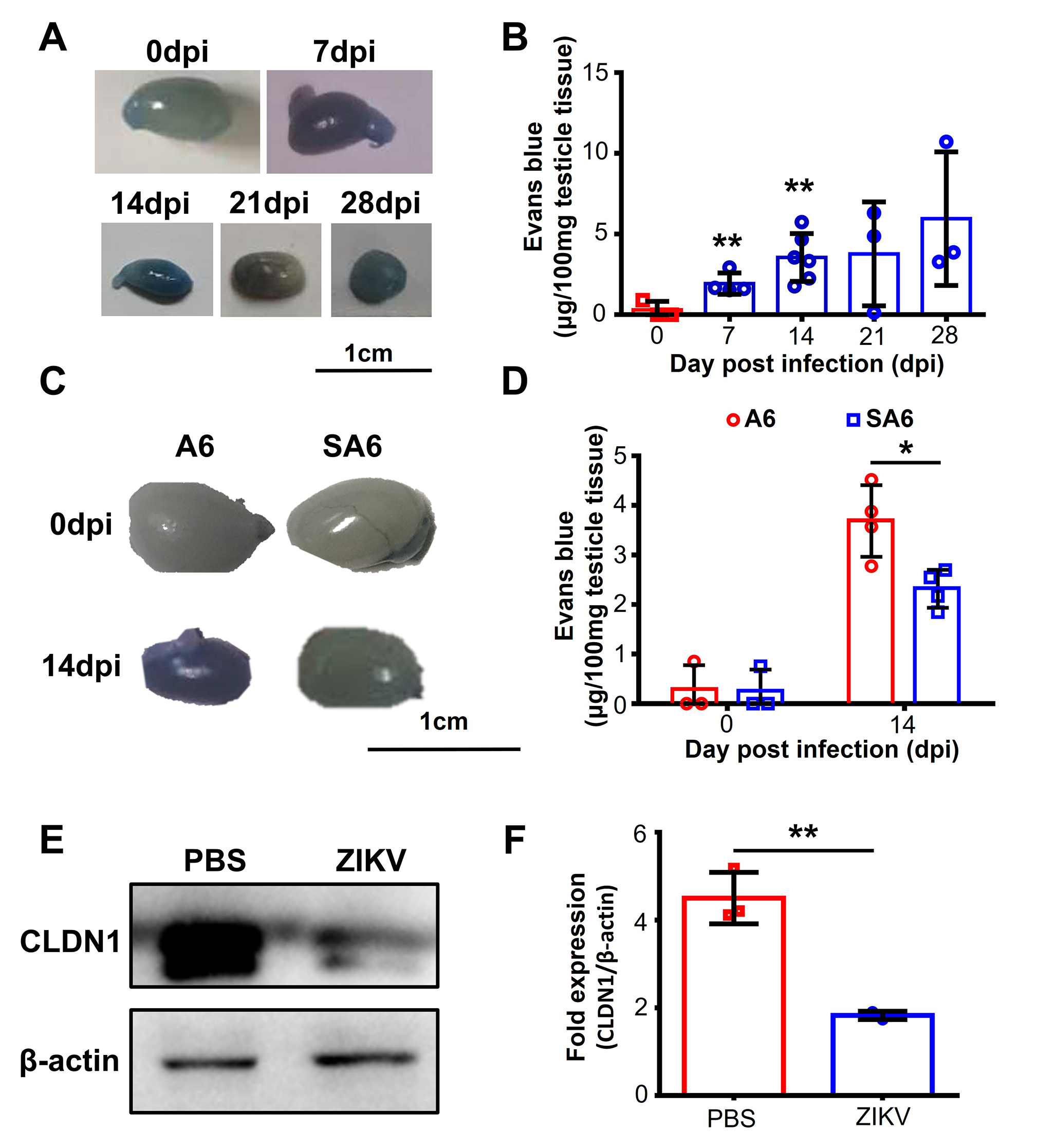

Supplement: S8 Fig — (A and B) Changes of BTB permeability in ZIKV-infected testes of A6 mice. ZIKV-infected A6 mice were injected with Evans blue (EB) at indicated time point. (A) Representative pictures of testes injected with EB. (B) EB concentration in testicular tissues was determined by spectrophotometry at 620 nm and shown as means ± SEM. (n = 3–6 mice for each time point). (C and D) ZIKV-infected A6 and SA6 male mice were injected with EB at 0 and 14 dpi. (C) Representative pictures of testes injected with EB. (D) EB concentration in testicular tissues was determined by spectrophotometry at 620 nm and shown as means ± SEM. (n = 3–4 mice for each time point). EB concentration in testicular tissues from ZIKV-infected A6 or SA6 male mice was analyzed using the Student’s t test. *p < 0.05, **p < 0.01. (E) Expression of CLDN1 in ZIKV-infected testes of A6 mice. Testes from PBS-injected or ZIKV-infected (14 dpi) A6 mice was subjected to Western blot with anti-CLDN1 or anti-β-actin antibody. (F) Fold change of CLDN1 was analyzed by Image J and shown as means ± SEM. (n = 3 mice for each group) and analyzed using the Student’s t test. *p < 0.05, **p < 0.01. (TIF) [file ppat.1009019.s009.tif]

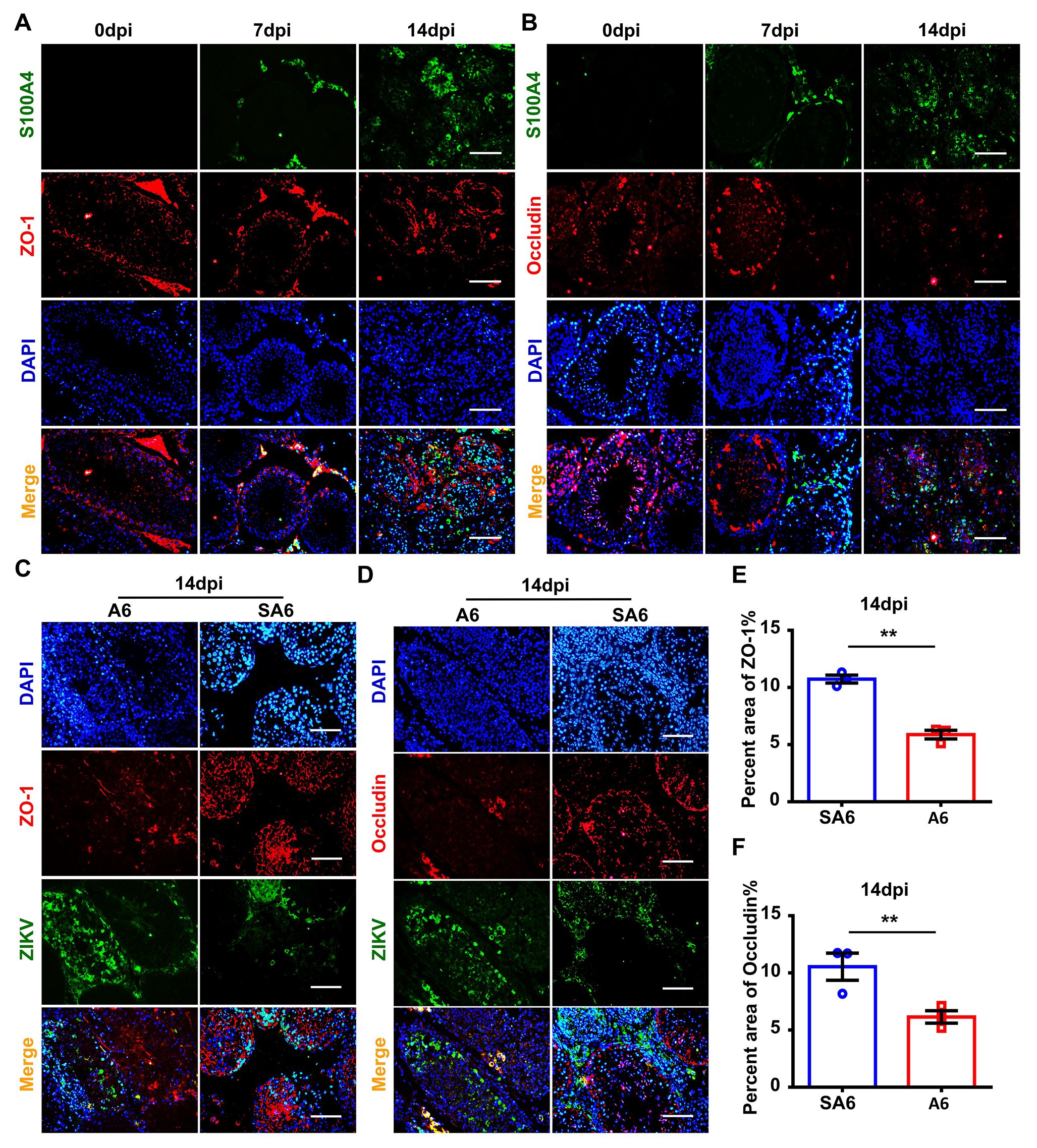

Supplement: S9 Fig — (A and B) Analysis of tight junction related proteins in ZIKV-infected testes. Testicular sections from ZIKV-infected A6 mice at indicated time points were analyzed by co-immunofluorescence staining with anti-S100A4 antibody and (A) anti-ZO-1 antibody or (B) anti-Occludin antibody. Nuclei were stained with DAPI. Scale bar, 25 μm. Related to Fig 6. (C and D) Testicular sections from ZIKV-infected A6 and SA6 mice at 14 dpi were analyzed by co-immunofluorescence staining with anti-ZIKV antibody and anti-ZO-1 antibody (C) or anti-Occludin antibody (D). Nuclei were stained with DAPI. Scale bar, 25 μm. (E and F) The expression of ZO-1 (E) and Occludin (F) in testes from ZIKV-infected A6 or SA6 male mice at 14 dpi were analyzed by calculating the area of red as a percentage of the total area by Image J and shown as means ± SEM (n = 3 mice for each group). Data were analyzed using the Student’s t test. *p < 0.05, **p < 0.01. (TIF) [file ppat.1009019.s010.tif]

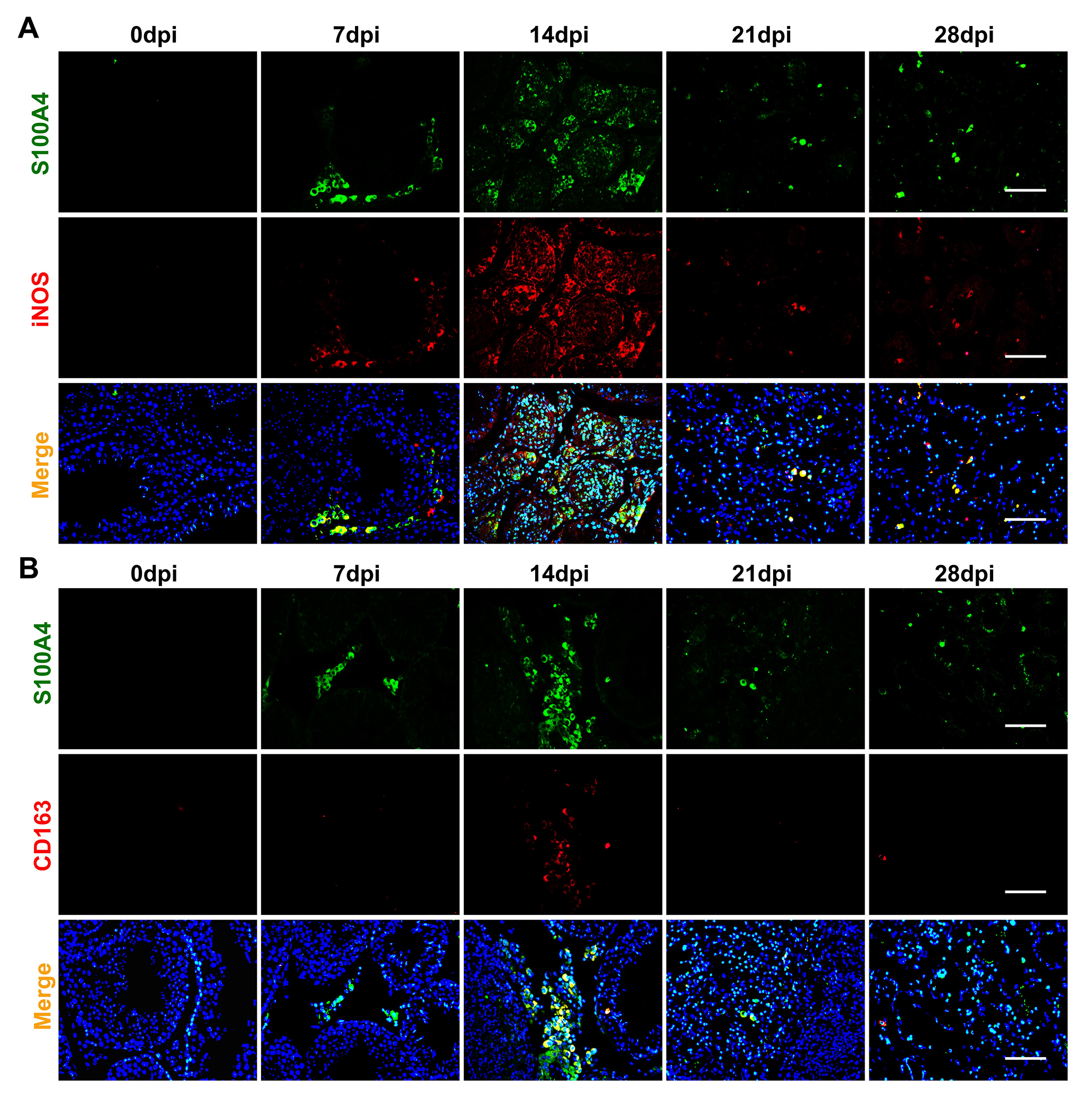

Supplement: S10 Fig — Testes from ZIKV-infected A6 mice were isolated at indicated time points and subjected to co-immunofluorescence staining with anti-ZIKV antibody and (A) anti-iNOS antibody, or (B) anti-CD163 antibody. Nuclei were shown with DAPI. Scale bar, 25 μm. The quantification of these results was shown in Fig 7B. (TIF) [file ppat.1009019.s011.tif]

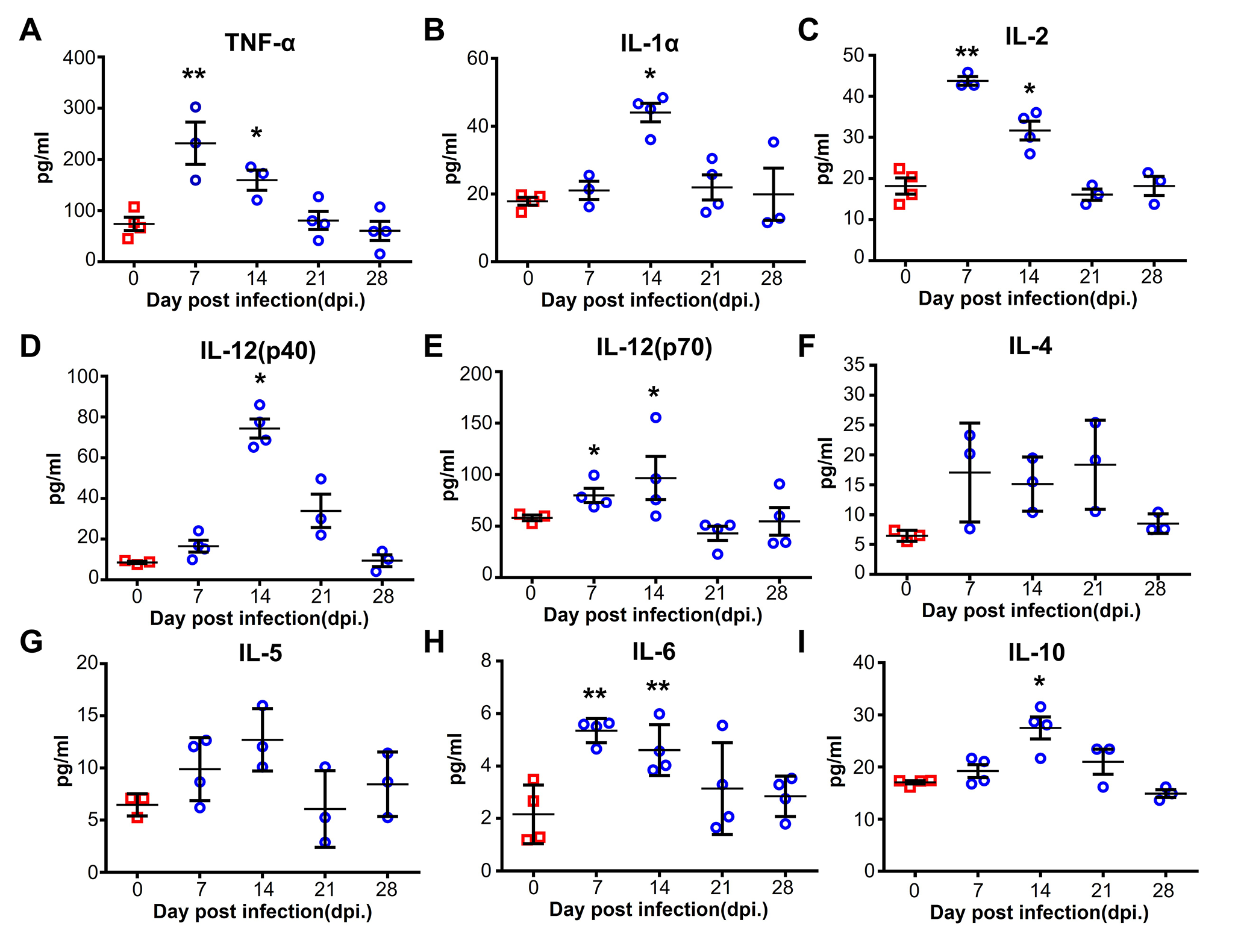

Supplement: S11 Fig — Expression of various cytokines in ZIKV-infected testes of A6 mice from 7 to 28 dpi was measured using Luminex assay and shown as means ± SEM. (n = 3–4 mice for each time point). All data were analyzed using the Student’s t test. *p < 0.05 versus 0 dpi, **p < 0.01 versus 0 dpi. (TIF) [file ppat.1009019.s012.tif]
