# Supplementary material for: S100A4+ macrophages facilitate zika virus invasion and persistence in the seminiferous tubules via interferon-gamma mediation
Source: PLoS Pathog. 2020 Dec 14;16(12):e1009019. doi: 10.1371/journal.ppat.1009019 (PMC7769614; doi:10.1371/journal.ppat.1009019)
Supplement: Attachment — Submitted filename: Response to reviewers PPATHOGENS-S-20-00984.docx [file ppat.1009019.s013.docx]

**Dear editors and reviewers**

Thank you very much for your kind consideration and comments on our manuscript. All comments and suggestions are constructive and very helpful to improve the manuscript. We carefully revised the manuscript by closely following your comments, adding new data and modifying the English. Main revisions include:

(1) Several new experiments were performed to address reviewers’ questions, and consequently the supplementary figures were reorganized, with 26 new panels added.

(2) Eight panels in figures were revised as requested by reviewers.

(3) A schematic diagram was added to help understanding the mechanism.

(4) Important issues suggested by reviewers were discussed or described in revised manuscript.

(5) A few words were changed with more suitable words according to reviewers’ comments.

(6) With the help of editors from AJE, grammar and syntax were carefully improved throughout.

The following are the point-to-point replies.

**Point-to-point replies:**

**Part I - Summary**

**Reviewer #1:** The manuscript titled “S100A4+ Macrophages Assist Zika Virus to Invade and Persist in Seminiferous Tubules via Interferon-gamma Mediation” describes the role of S100 A4 macrophages in testicles during ZIKV infection. The mechanism that regulates the ZIKV pathogenesis in infecting sperm cells has not been delineated yet. While the finding shed some new and important knowledge in this particular area, authors should emphasize the significance of their findings in terms of disease prevention.

**Reply:** Thank you for recognizing our research and providing valuable advice. The presence of ZIKV in semen and its damage to the male reproductive system is a major threat to human health. In this study, we proposed that S100A4+ macrophages facilitate ZIKV’s damage on the reproductive system. This finding shed new light on the pathogenic mechanism of ZIKV infection in testes and identified several factors such as S100A4 and IFN-γ as crucial factors, providing potential targets for disease prevention. Various strategies targeting S100A4 or IFN-γ have been developed and tried for diseased treatment. For example, recent studies have shown that S100A4 plays important roles in the pathogenesis of lateral sclerosis, and its inhibitor niclosamide slow the progression of the disease and reduces severity [1]. We wonder whether these strategies including the inhibitor may also have effects on ZIKV infection in testes, and we will investigate this hypothesis in subsequent studies.

We have discussed the significance of these findings in terms of disease prevention in the revised manuscript in lines 602-612.

**Reviewer #2:** Here, the authors have produced a paper in which the mechanisms of Zika virus testicular invasion and persistence were examined. The authors use a combination of in vivo and in vitro models to show that S1004a+ macrophages significantly contribute to the trafficking of ZIKV to the male reproductive tract and clearly play a crucial role in establishment of ZIKV infection in the testicles. The results are comprehensive, but I believe there are few things that need to be addressed to facilitate understanding by the reader and a few scientific issues that are not currently addressed. First, grammar and syntax need to be improved throughout. Second, there are no line numbers, which make it difficult to call out specific areas that need improvement. Please include line numbers. Specific comments follow.

**Reply:** Thank you for your positive comments and specific suggestions. To improve the manuscript, we revised the grammar and syntax throughout the text and added line numbers in the revised manuscript. The other comments are replied to point-to-point below.

**Reviewer #3:** The manuscript by Yang et al. presents data showing that S100A4+ macrophages are a target of ZIKV infection in the testes, where they are recruited. These cells secrete IFNgamma, which downregulates the tight junction protein Claudin-1 in the testes. S100A4+ cells may therefore increase the permeability of the blood-testes barrier. The experiments are performed well and identify a novel cell type infected by ZIKV in the testes.

**Reply:** Thank you for your encouragement.

**Reviewer #4:** In this study, the authors use A6 mice, AG6 mice and S100a4-/- Ifnar-/- mice to investigate the pathological changes in the ZIKV-infected testis. They report to find a subpopulation of S100A4+ bone marrow-derived macrophages in the testes of ZIKV-infected mice. They also found that S100A4+ macrophages were specifically recruited into interstitial space of the testes and differentiated into interferon-γ expressing M1 macrophages. Although **t**he study question is important, and most of the results are clearly presented, there are several weaknesses in the data interpretation and choice of the animal model.

**Reply:** Thank you for your positive comments and constructive suggestions. Specific comments including those about animal model will be replied below point-to-point.

Major weakness is the choice of animal model. During the early stages of animal model development for Zika, Ifnar-/- mouse were used to study ZIKV pathogenesis including testicular infection. However, very soon it was realized that these immunocompromised mouse models do not mimic human testicular infection of Zika virus. Persistence of ZIKV in human testes is not accompanied with pain or orchitis-like symptoms and severe testicular damage as seen in these AG6 or A6 mouse models. Therefore, any data on severe disruption of BTB and gross pathology seen in these mouse models is not applicable to human ZIKV infection and persistence in the testes and thus lowers the enthusiasm of this study. Recently immunocompetent mouse models including hSTAT2 knock in mice have been established to study ZIKV pathogenesis in placenta and fetal brains. They would be ideal to address the overall objectives of this study. These mice are available at commercial vendors and it will be nice if at least few experiments are repeated with immunocompetent mice to strengthen the relevance of this study.

**Reply：**Verifying the roles of S100A4+ macrophages in different mouse models is a valuable suggestion. To obtain the hSTAT2 knock in (KI) mice, we contacted The Jackson Laboratory as recommended by Prof. Michael Diamond who established the hSTAT2 KI mouse model. We are currently in contact with The Jackson Laboratory to arrange transport of hSTAT2 KI mice. The procedure may take more time than usual due to COVID-19. Once we obtain hSTAT2 KI mouse, we will design experiments to observe the effect of ZIKV infection on their testes.

Unlike other mosquito-borne flaviviruses, ZIKV can transmit sexually and vertically and persist in semen for a long time. These characteristics have been well observed in A6 and A129 mice, suggesting that the pathogenesis of ZIKV infection in these mice is similar to that in humans. Therefore, A6 and A129 mice are useful animal models for ZIKV infection and are widely used for ZIKV research. Of course, there are some limitations to using A6 and A129 mice for studying ZIKV infection because they are immunocompromised. For example, damage of testes induced by ZIKV in A6 mice was more severe than that in humans. However, testis tropism of ZIKV, long-term persistence in semen and lymphocyte infiltration are similar to those of humans. Thus, we believe that results obtained from the A6 mouse model provide beneficial data for us to understand the pathogenesis of ZIKV infection in humans. Very recently, Jamie et al. reported that there was macrophage infiltration in testes of ZIKV infected male olive baboons [2], which is consistent with the results obtained in A6 mice.

Although animal models that mimic human disease are important and ideal, there are few animal models that perfectly mimic human disease at this time. In many situations, mouse models provide a fast and affordable screening system that can quickly identify research targets. From this perspective, A6 mice which magnify ZIKV damage to the testes would be helpful for related follow-up studies.

Additionally, according to our long-term plan, we first used the more susceptible AG6 mice to confirm ZIKV damage in the testes and then switched to A6 mice, which usually survive much longer with ZIKV infection, to characterize the roles of S100A4 macrophages in testis damage induced by ZIKV infection. We plan to next use primate models to verify the effects of S100A4+ macrophages in ZIKV-induced testis damage. Our application “The characteristics and mechanism underlying testis damage induced by ZIKV infection in northern pig-tailed macaques, Reference number: U1902210” has been approved by the NSFC-Yunnan Joint Funding. With this funding, we will use pig-tailed macaques to investigate damage in the male reproductive system induced by ZIKV infection and the underlying mechanisms. Roles of S100A4+ macrophages will be the focus of our future research. The project is expected to be completed within two to three years, and we would be happy to share our data with researchers in the field at that time.

**Other comments:**

Fig 1: What is the ZIKV titers in other organs at these time points. Is the virus cleared from other organs and blood by day 14 and ZIKV has established persistence only in the immune privilege compartment of the testes? In the A6 mouse models, it is important to show this data to validate persistence. If virus is present in the blood and other organs at later time points, that will mean that immunocompromised mice cannot clear virus from all organs including testes and will affect data interpretation. The author’s previous AG6 data demonstrates severe damage of the testes while symptoms of testicular damage like orchitis and pain are not reported in humans. Since Fig 1 experiments use A6 mice (not AG6), the virus replication in blood and other organs at different time points and survival data should be included here to correlate with testes virus titers and gene expression data. Actually, a comparative figure of virus, and S100A4 and chemokine gene expression in testes and other organs like blood, brain and lung would strengthen the study and determine if the effect is specific to testes or a limitation of this animal model.

**Reply:** According to your comments, we used RT-qPCR to measure viral load in major organs of ZIKV-infected A6 mice; these data were added to the revised manuscript as **Supplemental** **Fig. 1A**. The new result showed that the viral load in the liver and lung peaked at 7 dpi, then gradually decreased and was completely cleared at 21 dpi. A similar trend was observed in the brain, but the reduction in viral load was slow and a small amount of virus was also detected at 21 dpi, which may be related to the immune-privileged environment of the brain. The viral load in whole blood showed a slow decreasing trend.

Notably, the viral load in testes was significantly higher than that in the liver, lung and brain after ZIKV infection, indicating the susceptibility of testes to ZIKV. It peaked at 14 dpi instead of 7 dpi and was much higher than that in other organs at 21 dpi and 28 dpi. These results suggested that ZIKV was completely cleared in most peripheral organs, but persistent infection was established in the immune privilege organs, such as the brain and testis, which was the most susceptible to ZIKV in A6 mice, consistent with your speculation.

The causal relationship between viremia and viral load in testes is an important issue. Your concern that the long-term presence of ZIKV in testes results from long-term presence of ZIKV in the blood. However, there was no significant difference in viremia at 14 dpi and 7 dpi, while the viral load in testes was significantly higher at 14 dpi than at 7 dpi. Combined with a significantly lower viral load in peripheral organs at 14 dpi but not at 7 dpi, we think that long-term viremia may be a comprehensive reflection of the changes of viral load in major organs including the testis, brain, lung, liver, etc. Additionally, we used whole blood instead of serum to measure viral RNA load, and this might be another reason to explain the long-term presence of viremia in our experiments.

We combined viral load, testis lesions, and survival rate of A6 mice for further analysis. Although the viral load in testes was different from that in other peripheral organs, it was consistent with S100A4+ expression and the number of S100A4+ macrophages in the testes. These results indicate that aggregation of S100A4+ macrophages in testes is an important factor underlying ZIKV's long-term existence in semen, which may be a distinct pathological characteristic of testes during ZIKV infection.

According your suggestion, we also measured levels of several chemokines in the testes at different time points. ZIKV-infected A6 mice demonstrated increased CCL2, CCL3 and CCL5 in the testes, and their changes were consistent with the dynamic of viral load and S100A4+ macrophage abundance in testes. These results were added as **Supplemental Fig. 2F-H** in the revised manuscript.

Fig 2E: doesn’t make sense- why would the virus titers go down at 36 hrs and then go up again? Was the virus at 24 hrs residual virus after infection? This needs clarification

**Reply:** Your consideration is right. We repeated this experiment and removed the residual virus as much as possible. **Fig. 2E** in the revised manuscript was updated with data obtained from new experiments, in which virus titers before 36 hours were lower but the tendency afterward was similar with that of the previous figure.

Fig 4: By day 14 the boundaries between seminiferous tubules (ST) and interstitium appears to be totally lost and the testicular damage is extensive. Therefore, it cannot be said that the virus infected cells **diffused** into ST. At that time, there is no distinct interstitium and ST lumen.

**Reply:** The seminiferous tubule and the interstitial boundary was destroyed at 14 dpi, but there are three reasons lead us to think that it may not completely disappear. First, as shown in **Fig. 2A**, the interstitial boundary and the seminiferous tubule were clearly observed at that time. Second, Sertoli cells and myoid epithelial cells around the tubules were clearly defined, as demonstrated in **Supplemental** **Fig. 4**. Finally, CD8+ T cells were excluded from the tubules at 14 dpi **(Fig. 3)**. These results suggested that there was still barrier structure around the seminiferous tubules, which could still exclude some cells such as CD8+ T cells until 21 dpi after infection.

However, to avoid any confusion, “diffuse” was replaced by “appeared” in line 275.

Last sentence of Fig 4 results-‘At late stage when spermatogenic cells diminished, luminal S100A4+ macrophages replaced them to support the infection of ZIKV. In this way, ZIKV changed target cells and persisted in testicles for longer time’- Here do the authors mean that ZIKV infected Spermatogonium stem cells die due to infection at later stages and therefore the virus is seen only in macrophages? In that case they should conduct caspase-3 or some cell death assays to determine death of infected spermatogonium cells.

**Reply:** Yes, we confirm that we observed the phenomenon that “luminal S100A4+ macrophages replaced spermatogenic cells to support the infection of ZIKV at the late phase of infection”.

Both immunofluorescence (IFA) with anti-DDX4 antibodies **(Fig. 4A)** and electron microscopy **(Fig. 4D-I)** confirmed that the spermatogenic cells decreased at 14 dpi. The results were consistent with previous reports by Ma W et al. and Govero J et al., both studies reported that spermatogenic cells decreased, leading to testis atrophy and sterility in mice after ZIKV infection [3,4].

Moreover, we further found that S100A4+ macrophages survived longer than spermatozoa in ZIKV-infected A6 testes. When spermatozoa disappeared, S100A4+ macrophages replaced them to support ZIKV replication. In particular, we showed two adjacent tubules containing different cells as target cells for ZIKV in **Fig. 4B**. The DDX4+ spermatogenic cells were target cells on the left tubule, while the S100A4+ macrophages supported ZIKV infection on the right tubule. This result may provide clear and convincing evidence supporting our conclusion.

According to your suggestion, we used testis sections from ZIKV-infected A6 mice to perform co-immunofluorescence staining with anti-DDX4 and anti-caspase-3 antibodies. Cells co-stained with anti-DDX4 and anti-caspase-3 antibodies were visible at 21 dpi, a very late phase of the infection, indicating that death of spermatogenic cells may not be caspase-3-mediated apoptosis. The specific mechanism involved in this process remains to be elucidated. This result was added to the revised manuscript as **Supplemental** **Fig. 5A-C**.

Fig 5A: Again, ZIKV viral load in other organs should be assessed in SA6 mice. Based on Fig 5A, it appears that virus in the blood is high in the A6 mice at day 7 and 14 and correlates with high virus in the testes. What is the virus titer in the blood and testes in both A6 and SA6 mice at day 7, 21 and 28. Does the kinetics of virus clearance in the blood and other organs follow similar pattern as in testes? Is less virus in SA6 mice in the testes at days 10 and 14 due to less virus in the blood? Can a simple interpretation of Fig 5A-J can be that since the virus reaching testes is less in SA6 mice (due to reduced viremia) that leads to reduced replication in the testes and reduced damage to the ST cells and spermatogonium cells.

**Reply:** As you suggested, we detected the changes of viral load in various organs of ZIKV-infected SA6 mice at 7 dpi and 14 dpi. Due to the restrictions during the COVID-19 epidemic, we were unable to obtain sufficient mice for other time points. Fortunately, the results at 7 dpi and 14 dpi reflected the most obvious changes in viral load. We inserted the data as **Supplemental** **Fig. 6F** in the revised manuscript and hope they could address the question.

The changes of viral load in the liver and brain of SA6 mice were similar to those in A6 mice. However, viremia was different from that in A6 mice; it was significantly lower at 14 dpi than at 7 dpi in SA6 mice **(Fig. 5A)**.

In both A6 and SA6 mice, the viral load in testes was higher than that in other tissues, indicating that depletion of S100A4+ macrophages did not affect testis susceptibility to ZIKV in the early stage of infection. However, the viral load in SA6 testes showed a different trend from that in A6 mice, in which the highest viral load was observed at 14 dpi. In contrast, viral load in SA6 testes peaked at 7 dpi **(Supplemental Fig. 6F)**, indicating that ZIKV could not invade into the seminiferous tubule and could not replicate continuously in the SA6 testes in the absence of S100A4+ macrophages **(Fig. 5D-L)**.

As mentioned above, the causal relationship between viremia and viral load in testes is an important issue. Drastically reduced viremia was observed in SA6 mice compared to testes, suggesting that viral load in testes may contribute to viremia, which is consistent with the results in A6 mice. However, due to the lack of S100A4+ macrophages in SA6 mice, ZIKV invasion to the seminiferous tubules was inhibited. Therefore, the viral load in the testes began to decrease at 7 dpi, when ZIKV could replicate continuously by virtue of S100A4+ macrophages entering the seminiferous tubules in A6 mice.

Although the authors measured IFN-g, since S100A4 macs are pro-inflammatory, it is pertinent to measure the cytokine/chemokine response in the testes using multiplex assays or ELISA (not RT-PCR). The kinetics of S100A4 positive macs and virus titers in the testes and its correlation with cytokines/chemokines produced at different time points and disruption of ST boundaries will be insightful.

**Reply:** Thank you for your comments. During revision, we used the Luminex assay to detect a panel of cytokines/chemokines in the testes of ZIKV-infected A6 mice. Chemokine data and cytokine data are shown in the revised manuscript as newly added **Supplemental** **Fig. 2F-H** and **Supplemental Fig. 11** and are discussed in line 203-206 and 437-445, respectively. The expression levels of pro-inflammatory cytokines including TNF-α, IL-1, IL-6, and IL-12 and the chemokines CCL2, CCL3, and CCL5 significantly increased from 7 dpi to 14 dpi. In contrast, the expression of anti-inflammatory cytokines, including IL-4 and IL-10, had no change or only slight changes. These results are consistent with M1-type polarization of S100A4+ macrophages.

**Part II – Major Issues: Key Experiments Required for Acceptance**

**Reviewer #1:** 1. If the bone marrow derived macrophages are not required during early stage of infection, how does the virus enters and cause infection and how it overcomes the host IFN response to cause an infection?

**Reply:** This is an important issue for ZIKV infection. ZIKV infection can cause viremia in both humans and mice, and dissemination following blood circulation is the likely source of early infection in testes. According to previous reports by us and other groups, ZIKV infection in testes initiated in the testicular interstitium and followed by spread into seminiferous tubules.

The testicular interstitium contains several types of cells including Leydig cells, peritubular myoid cells, endothelial cells, macrophages, dendritic cells (DC), T lymphocytes and mast cells. Our previous study found that testicular resident macrophages are an important target for ZIKV at the early stage [5]. The current study is a follow-up of our previous research, and we found that bone marrow-derived macrophages promote ZIKV infection and testis damage at the late phase of ZIKV infection.

Consistently, Konstantin et al. demonstrated that interstitial leukocytes are the initial targets of ZIKV in the early stage of infection, and they are necessary for downstream infection of the seminiferous tubules [6]. Taken together, we propose a relay during ZIKV infection in the testes in which ZIKV replicates in testicular resident macrophages in the very early stage, followed by Sertoli cells and spermatogenic cells, while S100A4+ macrophages are the last target cells at the late stage of ZIKV infection.

Type I interferon is an important antiviral molecule. However, ZIKV can overcome the host IFN response through multiple approaches to initiate infection. For example, ZIKV can inhibit STAT2 downstream of IFN-I signaling pathway in humans, but not in mice to antagonize production of IFN-I and ISGs; ZIKV NS2B3 protease can down-regulate STING-dependent antiviral cellular defenses [7,8].

In this study, we used A6 mice which are IFN-I receptor knockout mice that lack functional IFN-I signaling. To study the function of S100A4, we mated the S100A4 knockout mouse with A6 mouse and obtained the SA6 mouse. This allowed our animal model to bypass the type I interferon-mediated antiviral response and ensure that they were infected by ZIKV.

2. It was previously documented that testicular macrophages can be infected by ZIKV in vitro. These macrophages were reported to have a dual role by triggering host immune response and also increasing the permeability of BTB. According to the current findings, authors should discuss how the myeloid macrophages could overcome the immune response/immune suppressive properties of resident macrophages during ZIKV infection.

**Reply:** This is an interesting question. We briefly introduced testicular resident macrophages in the original manuscript. In general, testicular resident macrophages are M2 macrophages, and they establish an immunosuppressive microenvironment by expressing IL-10 or presenting spermatogonia auto-antigens. Immunofluorescence staining using anti-iNOS or anti-CD163 antibodies have showed that M1 macrophages became more abundant than M2 macrophages in ZIKV-infected testes. Based on the result, we speculated that the overwhelming M1 macrophages changed the M1/M2 balance in the testes and consequently altered the immune suppressive testicular environment.

To test this hypothesis, we measured a panel of cytokines in ZIKV-infected testes during the revision. In spite of a slight increase, the change fold of IL-10 (1.62) was lower than that of pro-inflammatory cytokines such as TNF-α (3.13), IL-1α (2.36), IL-6 (2.15) and IL-12(p40) (8.67). These results, which were added to the revised manuscript as **Supplemental Fig. 11**, suggested that although ZIKV infection in testes induced increases in both M1 and M2 macrophages, the M1 macrophages (S100A4+ macrophages) were overwhelming and thus overcame the immune suppressive properties of resident macrophages. The related discussion can be found in line 572-580 of the revised manuscript.

3. As this is the first report on role of s100A4 in ZIKV, the authors should discuss the significance of S100 A4 macrophages in any other viral infection (per say) that are sexually transmitted.

**Reply:** Thank you for the suggestion. We also reviewed the relevant reports. Alex and Perter reviewed the breadth of viruses in human semen and summarized 27 viruses that can be found in human semen, including human immunodeficiency virus (HIV), hepatitis B virus (HBV), hepatitis C virus (HCV), human papillomavirus (HPV), Epstein-Barr virus (EBV) and mumps virus et al. [9]. To our knowledge, no evidence has been reported regarding the involvement of S100A4+ macrophages in sexual transmission of these viruses.

However, it is worthy to mention that Zhu et al. found that the HBV HBx gene could up-regulates the expression of S100A4 and promotes the occurrence and development of liver cancer [10]. The authors believe that S100A4 may be one of the therapeutic targets for hepatocellular carcinoma caused by HBV. We have added this content to the Discussion section in line 502-511.

4. What would be the rationale for the recruitment of bone marrow macrophages during ZIKV infection; Would it be an unique process to ZIKA?

**Reply:** This is an interesting question. In the original manuscript, RNA-seq revealed enhanced expression of chemokines CCL2 and CCL5 in ZIKV-infected testes **(Fig. 1A)**, suggesting the S100A4+ macrophages might be recruited by these chemokines. During the revision, we further validated the production of these chemokines at the protein level, and added the results as **Supplemental Fig. 2F-H**. The Luminex assay showed that CCL2, CCL3 and CCL5 all significantly increased in ZIKV-infected testes. Of them, CCL5 nearly peaked at 7 dpi. Taken together, RNA-seq and the Luminex assay both suggested that ZIKV-infected testes released a large number of chemokines, which may play critical role in recruiting bone marrow macrophages into the testes.

In our study, obvious aggregation of S100A4+ macrophages during ZIKV infection was only observed in the testis and spleen **(Fig. 1 and Supplemental Fig. 1B-G)**. The spleen functions as reservoir for storage and rapid deployment of monocytes during inflammation [11]. The recruitment of S100A4+ macrophages was therefore a testis-specific process during ZIKV infection. However, in addition to ZIKV-infected testes, S100A4+ macrophages have also been observed at inflammatory sites for other pathogens and situations. Zhang et al. reported that S100A4+ macrophages increased in intestines infected with *Citrobacter rodents* and promoted the development of colitis by increasing its adhesion to intestinal epithelial cells [12]. Li et al. reported that S100A4+ macrophages play an important role in drug-induced liver cancer [13]. We speculate that S100A4+ macrophages may play roles in a variety of chronic infectious diseases; however, more studies are needed to address the question.

5. This study lack insights to the possible mechanism by which S100 A4+ cells support ZIKV infection into seminiferous tubules? It will be good to have this demonstrated.

**Reply:** To help understand the mechanisms proposed in our manuscript, we added a schematic diagram in **Fig. 8** of the revised manuscript. As illustrated in the schematic diagram, bone marrow-derived S100A4+ macrophages were recruited into the interstitial space of testes at the early stage of ZIKV infection in A6 mice. S100A4+ macrophages disrupted the integrity of the blood-testis barrier (BTB) by secreting IFN-γ which induced Claudin-1, a transmembrane protein crucial for BTB integrity, to translocate from the plasma membrane into nucleus, and thus invaded into seminiferous tubules, an immune-privileged environment which excludes CD8+ T cells. Being robust and susceptible to ZIKV infection, intraluminal S100A4+ macrophages supported prolonged ZIKV replication even when spermatogenic cells were eliminated at the late stage of ZIKV infection. **Fig. 8** was referenced in the Results section in line 470.

6. What would have been the role of newly recruited macrophages in sperm cells during cellular stress/tissue injury without ZIKV infection

**Reply:** This question is interesting. The roles of S100A4+ macrophages in testicular damage (not ZIKV infection) remain unknown. Nevertheless, macrophages have been reported to be involved in testicular damage. McDonald reported that, following vasectomy in human and many species, intraluminal macrophages in the testes destroy spermatozoa in the obstructed ducts, and degradation products are absorbed by the epididymal epithelium to form the sperm granuloma [14]. Melaine et al. injected Sendai virus through the scrotum and observed testes with immune-histological studies. They showed that the number of monocytes in the testicular stroma significantly increased, while the number of testicular macrophages remained unchanged, indicating that bone marrow-derived monocytes were recruited into the testes during Sendai virus infection; however, the function of these monocytes was not further investigated in this study [15]. Taken together, macrophage infiltration has been observed in many testicular diseases, but the molecular mechanisms underlying it remains elusive.

We have not investigated the effect of S100A4+ macrophages on sperm cells and testicular damages caused by factors other than ZIKV infection. We would like to test whether S100A4+ macrophages participate in other infectious diseases such as mumps caused by the mumps virus, which causes orchitis in some patients and may leads to infertility. The similarities between ZIKV and mumps virus may imply that S100A4+ macrophages play similarly important roles in the process.

7. It is essential to show the experiment of infecting S100A4 macrophage with ZIKV ex vivo and introduce the infected cells back to SA6 mice. Does seminiferous tubules invasion occur?

**Reply:** Adoptive transfer is an important experiment. However, we regret to inform you that these experiments cannot currently be performed due to the impact of COVID-19. We will carry out these experiments as soon as conditions permit.

**Reviewer #3:** 1. The authors say that the S100A4+ cells help ZIKV to evade CD8+ T cells. While they show that the S100A4+ cells are within the seminiferous tubules, while CD8+ T cells are excluded from the seminiferous tubules, this does not show that the S100A4+ cells help ZIKV to evade CD8+ T cells. In the S100A4 knockout mice, were CD8+ T cells increased in the seminiferous tubules?

**Reply:** We agree with you on this comment. The fact that S100A4+ macrophages facilitate ZIKV replication within the seminiferous tubules does not mean they were able to evade CD8+ T cells. Actually, when ZIKV-infected S100A4+ macrophages encountered CD8+ T cells, they were also killed by CD8+T cells **(Fig. 3B)**. The statement in the revised manuscript in line 238 was changed to “S100A4+ macrophages helped ZIKV to avoid CD8+ T immunity”.

As you suggested, we performed immunofluorescence to analyze the distribution of CD8+ T cells in ZIKV-infected SA6 testes at 14 dpi and quantified the number of CD8+ T cells inside or outside the seminiferous tubules using Image J. According to these results which have been added into the revised manuscript as **Supplemental Fig. 7C-D**, a number of CD8+ T cells appeared in ZIKV-infected SA6 testes at 14d pi; however, the majority of them accumulated in the interstitial space, and only a few could infiltrate into the seminiferous tubule. These results demonstrated that CD8+ T cells were still unable to enter seminiferous tubule in the absence of S100A4+ macrophages. According to these results, it was the barrier effect of seminiferous tubules, rather than protection of S100A4+ macrophages, that blocked CD8+ T cells from entering the seminiferous tubules.

2. What is the permeability of the testes in S100A4 knockout mice after ZIKV infection?

**Reply:** As you suggested, we measured testicular permeability and analyzed the distribution of tight junction proteins ZO-1 and Occludin in ZIKV-infected testes of SA6 mice at 14 dpi. These results were inserted into the revised manuscript as **Supplemental Fig. 8C-D** and **Supplemental Fig. 9C-F**. The Evans Blue leakage assay revealed that ZIKV-infected SA6 testes had a lower permeability than those in A6 mice at 14 dpi **(Supplemental Fig. 8C-D)**. Accordingly, immunofluorescence staining showed that ZO-1 and Occludin expression was significantly higher in ZIKV-infected SA6 testes and was mostly localized within the seminiferous tubules, which is a pattern similar to that of normal testes **(Supplemental Fig. 9C-F)**. These results suggested that the depletion of S100A4+ macrophages in A6 mice attenuated testicular damages cause by ZIKV infection.

**Part III – Minor Issues: Editorial and Data Presentation Modifications**

**Reviewer #1: 1.** What makes the virus attach to the sperm for several months after ZIKV infection when S100 A4 starts decline 21dpi (Fig 2E)

**Reply:** This is the question that we ultimately want to answer. Yes, ZIKV in semen can persist for 180 days in humans, which is much longer than in mice. However, the findings in this study are all from ZIKV-infected mice, which cannot be directly equated with humans.

As for differences in the association of S100A4+ macrophages and persistence of ZIKV in semen between human and mice, there are several explanations: (1) in humans, S100A4+ macrophages infiltrate and disappear more slowly than in mice and may last longer; (2) in humans, S100A4 macrophages characteristics may not be exactly the same as those in A6 mice; and (3) other factors are possibly involved in ZIKV persistence in semen that we have not yet elucidated.

As a follow-up to this work, we will verify the roles of S100A4+ macrophages using primate models. Our application “The characteristics and mechanism underlying testis damage induced by ZIKV infection in northern pig-tailed macaques, Reference number: U1902210 (Reference number: U1902210)” was approved in 2019 by NSFC-Yunnan Joint Funding. This funding supports our investigation of male reproductive system damage induced by ZIKV infection and its underlying mechanisms. Roles of S100A4+ macrophages are a crucial focus in the plan. The study is expected to be completed in the next 2 to 3 years, and we would be happy to share our data with researchers in the field at that time.

2. Why there is an upregulation of macrophages in spleen during ZIKV. What is their role in tissue tropism?

**Reply:** The macrophages upregulation in the spleen during ZIKV infection is possibly due to the role of spleens in regulating monocyte circulation. Filip et al. reported that bona fide undifferentiated monocytes reside in the spleen and outnumber their equivalents in circulation. In response to ischemic myocardial injury, splenic monocytes increase their motility, exit the spleen and are recruited to injured tissue [11]. This finding suggests that spleens function as reservoirs for storage and rapid deployment of monocytes and that splenic monocytes are an important resource that the body exploits to regulate inflammation.

In addition to ZIKV infection, macrophage upregulation in the spleen has also been observed in a variety of infectious diseases and pathological conditions including Trypanosoma rhodesiense infection [16], very virulent infectious bursal disease viral infection [17], severe fever with thrombocytopenia syndrome viral infection [18] and experimental stroke in mice [19].

3. What would be the clinical relevance of serum claudin 1 and ZIKA-can it be used for prognosis?

**Reply:** Although serum Claudin-1 has been recently proposed as a potential diagnostic index for melanoma and colorectal cancer [20,21], there is little evidence showing the association between serum Claudin-1 and infectious diseases including ZIKV.

However, it is a promising and intriguing idea. Many vector-borne viral diseases are characterized by hemorrhagic fever and encephalitis, so that this idea is interesting not only for ZIKV, but also for some other vector-borne viruses. If possible, we will collect serum from flaviviruses-infected patients in the future and analyzed the clinical relevance of serum Claudin-1 and the severity of viral infection.

4. If possible, EM pictures (Fig 4D-I) will be easier to interpret with the same magnifying power and a simplified cartoon showing anatomical location of macrophage invading seminiferous tubules.

**Reply:** Thank you for the suggestion. We revised the scale bar of **Fig 4D-I** to unify the magnifying power.

A schematic diagram was added into the revised manuscript to demonstrate the accumulation and invasion of S100A4+ macrophages in ZIKV-infected testis **(Fig. 8)**.

5. The comparison between A6 and AG6 susceptibility (IFN-gamma effect) to ZIKV (Fig 7H- J) would be clearer with the arrows to point out the affected cells, the brightness of the staining colors are poorly presented.

**Reply:** Thank you for the suggestion. We revised **Fig7. H-J** and used arrows to point out the affected cells. The brightness of staining colors was also slightly adjusted for improved presentation.

**Reviewer #2:** **Introduction:** Aren’t A6 and AG6 mice also IFN-beta deficient?

**Reply:** Yes, A6 and AG6 are deficient in IFNAR, which is recognized by both IFN-α and IFN-β. To avoid confusion, the description in line 108-110 “However, in AG6 mice lacking interferon (IFN)-α and -γ receptors,” has been corrected to “However, in AG6 mice lacking interferon (IFN)-α/β and -γ receptors,”.

Results:

It would be helpful to know what timepoint RNAseq was done to understand when S10004a transcript abundance was increased.

**Reply:** Testes used for RNA-seq were harvested from ZIKV-infected mice at 5 dpi. S100A4 expression change was further validated using testes obtained at 7, 14, 21 and 28 dpi. This information was added to the revised manuscript in line 137.

Some strains of ZIKV cause rapidly fatal infection in type I interferon deficient mice at 10e4 PFU. Mice here survived until 28 days. Is this unusual? Also, why was d7 chosen as the earliest timepoint? Surely, invasion of the male reproductive tract occurs much earlier after establishment of infection in the periphery.

**Reply:** Although some ZIKV strains cause rapidly fatal infection in type I interferon deficient mice at 10e4 PFU, ZIKV-infected A6 mice (C57BL6-based type I interferon receptor deficient mice) had a consistent survival rate at 10e4 PFU in our lab, and most A6 mice survived the ZIKV infection **(Supplemental Fig. 6E, line with blue circles)**. In contrast, our previous work showed that AG6 mice (both type I interferon and type II interferon receptor deficient mice) all died upon infection with ZIKV even at lower doses [5]. We suspect that the survival rate of type I interferon-deficient mice after ZIKV infection may vary depending on virus strain and mouse background.

We chose 7 dpi as the earliest time point because: (1) ZIKV-infected A6 mice lost the most body weight during 5 to 10 dpi **(Supplemental Fig 6C, line with blue circles)**; and (2) ZIKV-infected testes did not display obvious pathological changes until 7 dpi **(Fig. 2A)**.

Figure 1: How was Zika virus infection confirmed in samples that were used for RNAseq? What are the ZIKV viral titers in testis samples at 7, 14, and 28 dpi?

**Reply:** Prior to RNA-seq, we used RT-qPCR to measure the ZIKV load in testes to make sure that all testes used for RNA-seq had similar levels of ZIKV RNA.

As you and reviewer 1 suggested, we measured the dynamics of viral load in testis and added the results as **Supplementary Fig. 1A** in the revised manuscript.

We also detected viral loads in other major organs. The viral load in the liver and lung peaked on day 7, then gradually decreased and was completely cleared on day 21. A similar trend was observed in the brain, but the reduction in viral load was slow and a small amount of virus was also detected at 21 days, which may be related to the immune-privileged environment of the brain. The viral load in whole blood showed a slow decreasing trend.

Similarly, we also detected the viral loads in testes and other organs of ZIKV-infected SA6 mice at 7 dpi and 14 dpi and added the results as **Supplementary Fig. 6F** in the revised manuscript. As shown in **Fig. 5A** and **Supplemental Fig. 6F**, the testis had a higher viral load than any other organs. Taken together, the testis is most susceptible to ZIKV in both A6 and SA6 mice.

Figure 5: numerous studies have shown clearance of ZIKV from the peripheral blood of type I interferon deficient mice by day 7. Here, you show detectable vRNA loads at 7 and 14 dpi. Is this consistent with previous work with this particular ZIKV strain in mice?

**Reply:** ZIKV used in our experiments is strain SMGC-1 (GenBank accession number: KX266255) which was isolated in Shenzhen, China from an imported ZIKV patient in 2016. The manifestations in A6 and AG6 mice infected with the strain have been consistent in our experiments since we obtained the strain in 2016.

As mentioned above, RT-qPCR revealed that while ZIKV RNA persisted in the testis and brain, it was completely cleared from peripheral organs such as the lung and liver after 14 dpi. These results suggested that viral persistence was a phenomenon specific to testes. Persistent viremia might be related to the average level of that in testis and other organs. Additionally, we used the whole blood instead of serum to measure viral RNA load, and this might be another reason to explain the long-term presence of viremia in our experiments. Similar to our observation, the prolonged duration of ZIKV viremia was observed to some extent in different mouse models and ZIKV strains [4,22], and it was not a phenomenon uniquely observed in our lab.

Figure 5: Given that serum and testis viral loads were broadly similar, albeit lower in the SA6 group it would be interesting to know if SA6 have an equivalent capacity to sexually transmit ZIKV and/or how much infectious virus is shed in the semen compared to A6 mice.

**Reply:** We agree with you that it would be interesting to test the capacity of ZIKV-infected mice for sexual transmission of ZIKV. We tried to mate ZIKV-infected A6 mice with healthy female mice, but failed to establish a sexual transmission model, possibly due to infection in general and the severe testicular damaged.

Instead, we measured ZIKV RNA in semen from ZIKV-infected A6 and SA6 mice at 10 dpi, and added the results as **Supplemental Fig. 6G** in the revised manuscript. RT-qPCR results revealed that A6 mice had significantly higher ZIKV RNA levels than SA6 mice **(Supplemental Fig. 6G)**, suggesting that in the absence of S100A4+ macrophages, ZIKV replication in the testis was reduced.

In general, why was the IP route chosen for mouse inoculations and could this ultimately impact trafficking to the male reproductive tract compared to a subcutaneous inoculation, for example?

**Reply:** To study effects of ZIKV infection on the testis, Jennifer et al. challenged mice with ZIKV through subcutaneous administration in the footpad [4], while Ma et al. and Sofie et al. administered intraperitoneal ZIKV into mice [3,23]. Both routes were successful at establishing a testis-damaged model of ZIKV-infected mice. Compared with the subcutaneous route, the intraperitoneal is easier to perform, so we selected it for our experiments.

**Methods:**

More information is needed on the provenance of the virus strain used. What is the exact passage history of the virus isolate and stock used for experiments? Was the stock sequence verified and if so, what differences (if any) exist between it and the GenBank sequence?

**Reply:** ZIKV used in our experiments is strain SMGC-1 (GenBank accession number: KX266255) which was isolated in Shenzhen from an imported ZIKV patient in 2016. Dr. George F. Gao (Institute of Microbiology, Chinese Academy of Sciences, Beijing, China) kindly provided us the strain soon after it was isolated. When we received the strain, we propagated it in C6/36 cells, verified the sequence and stored them in batches. Sequencing results at that time showed no difference from the GenBank sequence.

The virus used from that point forward was propagated from the stock. Each vial in the stock was propagated for less than 10 times to avoid accumulation of mutations, but was not sequenced every time. The passage number was 6 to 8 in the current study.

**Reviewer #3:** 1. Some editing of grammar is required.

**Reply:** To improve English language, the revised manuscript has been reformatting and corrected by professional experts.

2. A graphical depiction of the proposed mechanism may be helpful.

**Reply:** Thank you for the suggestion. To help to understand the mechanisms proposed in our manuscript, we added a schematic diagram in revised manuscript to show the roles of S100A4+ macrophages in ZIKV-infected testis in A6 mice **(Fig. 8)**.

**Reviewer #4: Minor comments:**

Introduction, first para, line 6- sentence requires reformatting and more specific details such as ‘some of them more than 6 months’ is vague- specific percentage or study details should be included.

**Reply:** The statement you mentioned was revised to be: A prospective study of 184 ZIKV infected men, showed that viral RNA is commonly present in the semen (60/184) and persists in one patient for more than 6 months. (Line 70-72)

Line 8 in the same paragraph- ‘because of threat to male health’- please elaborate on the data to support threat to the male health

**Reply:** The detailed data was added to line 73-78 in revised manuscript: “For example, a prospective observational study of 15 male volunteers with acute ZIKV infection revealed that total sperm count was significantly reduced from a median 119 × 10^6^ spermatozoa at day 7 to 45.2 × 10^6^ at day 30 [24]; a cohort study including 11 symptomatic men demonstrated that 11 of them had presence of leukocytes, 10 showed haematospermia and six showed oligospermia [25]”.

Testicle is wrong term at many places including Blood-Testicle-Barrier. Testicle generally refers to testes along with epididymis.

**Reply:** Thank you for the correction. Testicle was replaced with testis in places where it was misused.

Similarly, there are many sentences that need careful reading and either reformatting or inclusion of specific details.

English language and grammar should be carefully edited.

**Reply:** To improve English language, the revised manuscript has been reformatting and corrected by professional experts.

**References**

1	Serrano A, Apolloni S, Rossi S, Lattante S, Sabatelli M, Peric M, *et al.* (2019) The S100A4 Transcriptional Inhibitor Niclosamide Reduces Pro-Inflammatory and Migratory Phenotypes of Microglia: Implications for Amyotrophic Lateral Sclerosis. *Cells* **8**(10), 1261. https://doi.org/10.3390/cells8101261 PMID: 31623154

2	Peregrine J, Gurung S, Lindgren MC, Husain S, Zavy MT, Myers DA, *et al.* (2019) Zika Virus Infection, Reproductive Organ Targeting, and Semen Transmission in the Male Olive Baboon. *J Virol* **94**(1), e01434-19. <https://doi.org/10.1128/JVI.01434-19> PMID: 31597777

3	Ma W, Li S, Ma S, Jia L, Zhang F, Zhang Y, *et al.* (2016) Zika Virus Causes Testis Damage and Leads to Male Infertility in Mice. *Cell* 167: 1511-1524. [https://doi.org/10.1016/j.cell.2016.11.016 PMID:27884405](https://doi.org/10.1016/j.cell.2016.11.016%20PMID:27884405)

4	Govero J, Esakky P, Scheaffer SM, Fernandez E, Drury A, Platt DJ, *et al.* (2016) Zika virus infection damages the testes in mice. *Nature* **540**(7633):438-442. https://doi.org/10.1038/nature20556 PMID: 27798603

5	Sheng ZY, Gao N, Wang ZY, Cui XY, Zhou DS, Fan DY, *et al.* (2017) Sertoli Cells Are Susceptible to ZIKV Infection in Mouse Testis. *Front Cell Infect Microbiol* **7**:272. https://doi.org/10.3389/fcimb.2017.00272 PMID: 28680856

6	Tsetsarkin KA, Acklin JA, Liu G, Kenney H, Teterina NL, Pletnev AG, *et al.* (2020) Zika virus tropism during early infection of the testicular interstitium and its role in viral pathogenesis in the testes. *PLoS Pathog* **16**(7):e1008601. https://doi.org/10.1371/journal.ppat.1008601 PMID: 32614902

7	Grant A, Ponia SS, Tripathi S, Balasubramaniam V, Miorin L, Sourisseau M, *et al.* (2016) Zika Virus Targets Human STAT2 to Inhibit Type I Interferon Signaling. *Cell Host Microbe* **19**(6):882-90. https://doi.org/10.1016/j.chom.2016.05.009 PMID: 27212660

8	Ding Q, Gaska JM, Douam F, Wei L, Kim D, Balev M, *et al.* (2018) Species-specific disruption of STING-dependent antiviral cellular defenses by the Zika virus NS2B3 protease. *Proc Natl Acad Sci U S A* **115**(27):E6310-E6318. https://doi.org/10.1073/pnas.1803406115 PMID: 29915078

9	Salam AP, Horby PW. (2017) The Breadth of Viruses in Human Semen. *Emerg Infect Dis* **23**(11):1922-1924. https://doi.org/10.3201/eid2311.171049 PMID: 29048276

10	Zhu K, Huang W, Wang W, Liao L, Li S, Yang S, *et al.* (2018) Up-regulation of S100A4 expression by HBx protein promotes proliferation of hepatocellular carcinoma cells and its correlation with clinical survival. *Gene* **30**;749:144679. https://doi.org/10.1016/j.gene.2020.144679 PMID: 32330536

11	Swirski FK, Nahrendorf M, Etzrodt M, Wildgruber M, Cortez-Retamozo V, Panizzi P, *et al.* (2009) Identification of splenic reservoir monocytes and their deployment to inflammatory sites. *Science* **325** (5940):612-616. https://doi.org/10.1126/science.1175202 PMID: 19644120

12	Zhang J, Jiao Y, Hou S, Tian T, Yuan Q, Hao H, *et al.* (2017) S100A4 contributes to colitis development by increasing the adherence of Citrobacter rodentium in intestinal epithelial cells. *Sci Rep* **7**(1):12099. https://doi.org/10.1038/s41598-017-12256-z PMID: 28935867

13	Li Y, Wang J, Song K, Liu S, Zhang H, Wang F, *et al.* (2020) S100A4 promotes hepatocellular carcinogenesis by intensifying fibrosis-associated cancer cell stemness. *Oncoimmunology* **9**(1):1725355. https://doi.org/10.1080/2162402X.2020.1725355 PMID: 32117590

14	McDonald SW. (2000) Cellular responses to vasectomy. *Int Rev Cytol* **199**:295-339. https://doi.org/10.1016/s0074-7696(00)99006-5 PMID: 10874581.

15	Melaine N, Ruffault A, Dejucq-Rainsford N, Jégou B. (2003) Experimental inoculation of the adult rat testis with Sendai virus: effect on testicular morphology and leukocyte population. *Hum Reprod* **18**(8):1574-9. <https://doi.org/10.1093/humrep/deg323> PMID: 12871865.

16	Nagle RB, Dong S, Guillot JM, McDaniel KM, Lindsley HB. (1980) Pathology of experimental African trypanosomiasis in rabbits infected with Trypanosoma rhodesiense. *Am J Trop Med Hyg* **29**(6):1187-95. <https://doi.org/10.4269/ajtmh.1980.29.1187> PMID: 7446810.

17	Rasoli M, Yeap SK, Tan SW, Roohani K, Kristeen-Teo YW, Alitheen NB, *et al.* (2015) Differential modulation of immune response and cytokine profiles in the bursae and spleen of chickens infected with very virulent infectious bursal disease virus. *BMC Vet Res* **25**;11:75. https://doi.org/10.1186/s12917-015-0377-x PMID: 25884204

18	Jin C, Liang M, Ning J, Gu W, Jiang H, Wu W, *et al.* (2012) Pathogenesis of emerging severe fever with thrombocytopenia syndrome virus in C57/BL6 mouse model. *Proc Natl Acad Sci U S A* **109**(25):10053-8. https://doi.org/10.1073/pnas.1120246109 PMID: 22665769

19	McCulloch L, Alfieri A, McColl BW. (2018) Experimental Stroke Differentially Affects Discrete Subpopulations of Splenic Macrophages. *Front Immunol* **22**;9:1108. https://doi.org/10.3389/fimmu.2018.01108 PMID: 29872438

20	Karabulut M, Alis H, Bas K, Karabulut S, Afsar CU, Oguz H, *et al.* (2015) Clinical significance of serum claudin-1 and claudin-7 levels in patients with colorectal cancer. *Mol Clin Oncol* **3**(6):1255-1267 <https://doi.org/10.3892/mco.2015.626> PMID: 26807230

21	Tas F, Bilgin E, Erturk K, Duranyildiz D.(2016) Clinical significance of serum claudin-1 levels in melanoma patients. *Melanoma Res* **26**(4):377-81. https://doi.org/10.1097/CMR.0000000000000241 PMID: 26886788.

22	Larocca RA, Mendes EA, Abbink P, Peterson RL, Martinot AJ, Iampietro MJ, *et al.* (2019) Adenovirus Vector-Based Vaccines Confer Maternal-Fetal Protection against Zika Virus Challenge in Pregnant IFN-αβR-/- Mice. *Cell Host Microbe* **26**(5):591-600.e4. https://doi.org/10.1016/j.chom.2019.10.001 PMID: 31668877

23	Jacobs S, Delang L, Verbeken E, Neyts J, Kaptein SJF. (2019) A Viral Polymerase Inhibitor Reduces Zika Virus Replication in the Reproductive Organs of Male Mice. *Int J Mol Sci* **20**(9):2122. https://doi.org/10.3390/ijms20092122 PMID: 31032814

24	Joguet G, Mansuy JM, Matusali G, Hamdi S, Walschaerts M, Pavili L, *et al.* (2017) Effect of acute Zika virus infection on sperm and virus clearance in body fluids: a prospective observational study. *Lancet Infect Dis* **17** (11): 1200-1208. <https://doi.org/10.1016/S1473-3099(17)30444-9> PMID: 28838639.

25	Huits R, De Smet B, Ariën KK, Van Esbroeck M, Bottieau E, Cnops L. (2017) Zika virus in semen: a prospective cohort study of symptomatic travellers returning to Belgium. *Bull World Health Organ* **95** (12): 802-809. https://doi.org/10.2471/BLT.17.181370 PMID: 29200521
